# Novel Phosphonium-Based Ionic Liquid Electrolytes for Battery Applications

**DOI:** 10.3390/molecules27154729

**Published:** 2022-07-24

**Authors:** Andreas Hofmann, Daniel Rauber, Tzu-Ming Wang, Rolf Hempelmann, Christopher W. M. Kay, Thomas Hanemann

**Affiliations:** 1 Institute for Applied Materials, Karlsruhe Institute of Technology, Hermann-von-Helmholtz-Platz 1, D-76344 Eggenstein-Leopoldshafen, Germany; uvdyf@student.kit.edu (T.-M.W.); thomas.hanemann@kit.edu (T.H.); 2 Department of Chemistry, Saarland University, Campus B2.2, 66123 Saarbrücken, Germany; daniel.rauber@uni-saarland.de (D.R.); r.hempelmann@mx.uni-saarland.de (R.H.); christopher.kay@uni-saarland.de (C.W.M.K.); 3 London Centre for Nanotechnology, Bloomsbury Campus, University College London, 17-19 Gordon Street, London WC1H 0AH, UK; 4 Department of Microsystems Engineering, University of Freiburg, Georges-Köhler-Allee 102, D-79110 Freiburg, Germany

**Keywords:** ionic liquid, phosphonium, electrochemistry, batteries

## Abstract

In this study, we address the fundamental question of the physicochemical and electrochemical properties of phosphonium-based ionic liquids containing the counter-ions bis(trifluoromethanesulfonyl)imide ([TFSI]^−^) and bis(fluorosulfonyl)imide ([FSI]^−^). To clarify these structure–property as well as structure–activity relationships, trimethyl-based alkyl- and ether-containing phosphonium ILs were systematically synthesized, and their properties, namely density, flow characteristics, alkali metal compatibility, oxidative stability, aluminum corrosivity as well as their use in Li-ion cells were examined comprehensively. The variable moiety on the phosphonium cation exhibited a chain length of four and five, respectively. The properties were discussed as a function of the side chain, counter-ion and salt addition ([Li][TFSI] or [Li][FSI]). High stability coupled with good flow characteristics were found for the phosphonium IL [P1114][TFSI] and the mixture [P1114][TFSI] + [Li][TFSI], respectively.

## 1. Introduction

Due to their intrinsic properties with regard to ion content, ion mobility [1], states of aggregation [2], vapor pressure [3], volatility [4] and solubility properties [2], ionic liquids (IL) represent a class of substances that are already being used or intensively studied in many areas of application. Depending on the IL cation and IL anion employed, the properties can be controlled and specifically influenced over a very wide range. This makes ILs a versatile liquid material. Despite this, ILs are typically more expensive than competitive organic compounds, so consideration of the benefits and costs must be made in particular here. ILs can be classified in very different ways, with a simple classification based on the cation and anion classes they contain.

A subclass of phosphorus-containing ILs is the substance class of phosphonium-based ionic liquids which, in general, contain the cation [PR_1_R_2_R_3_R_4_]^+^ with the moieties R_1_, R_2_, R_3_ and R_4_ (e.g., R = alkyl, alkoxy, aryl, etc.) [5,6]. These are closely related to ammonium-based ILs, which, in contrast to phosphonium ILs, contain nitrogen as a center in the cation and have already been studied more extensively [7,8]. However, it has been shown that alkyl-substituted, as well as alkoxy-substituted phosphonium ILs, have promising physical properties [9], electrochemical properties [9,10,11], good oxidative stability [12] as well as favorable behavior in Li plating–stripping experiments related to ammonium ILs (higher Coloumbic efficiency) [13] for use in energy storage devices and are therefore currently being studied in detail [5]. Likewise, conducting salts can already be dissolved at concentrations ≥ 0.75 M, such as lithium bis(trifluoromethanesulfonyl) imide ([Li][TFSI]) [9,12] and lithium bis(fluorosulfonyl) imide ([Li][FSI]) [14]. Moreover, Al corrosion phenomena, which are often observed in [TFSI]^−^ containing electrolytes at potentials exceeding 3.7 V vs. Li/Li^+^ [15,16], have been shown to be suppressed in binary phosphonium IL–[Li][TFSI] systems at voltages beyond 4 V [17].

These properties make phosphonium IL compounds attractive for energy storage applications (e.g., batteries and supercapacitors) that require electrochemical stability to higher voltages (>3 V) [8,18,19]. Although ammonium-based ionic liquids are prepared from cost-efficient amines compared to the trialkyl phosphines, the resulting phosphonium ionic liquids show a range of favorable properties compared to the ammonium analogues. These usually include lower melting temperatures and improved conductivity [20,21,22], which expands possible applications and increases the performance of the resulting batteries. The synthesis of the phosphonium ionic liquids must be conducted under an inert atmosphere to avoid oxidation to the phosphine oxides but has the advantage of faster reaction rates and lower side reactions due to the higher nucleophilicity and lower basicity of the phosphines in comparison to the amines.

An application at high voltages (5 V vs. Li/Li^+^) with [methyl tributylphosphonium][TFSI] ionic liquid, which was used up to 20 wt.-% in carbonate-based electrolyte, indicates sufficient Al resistance (oxidative stability) even at very high end-of-charge voltages up to 5 V [23]. A similar finding was discovered for alkyl-substituted phosphonium ILs with [TFSI]^−^ [12] or [FSI]^−^ [14] as a counter ion while using analogous Li-containing conducting salts (i.e., [Li][TFSI] or [Li][FSI]) up to 5V without additional solvents. The application of phosphonium IL in gels for energy storage applications was also discussed [24]. Likewise, promising properties are shown already, although the mobility is limited by the polymer crosslinking and the systems thus require higher temperatures (oxidation stability up to 4 V, ionic conductivity at *T* = 100 °C of *κ* = 0.13 mS/cm) [24].

Triethyl-substituted phosphonium ILs, e.g., [P2225]^+^ and [P222(2O1)]^+^, have been studied sporadically in the past [9,13,25,26], but up to now, the variation of alkyl moieties (e.g., methyl) and the change of side chain length (butyl, pentyl) have been studied in detail only for ammonium-based systems and cannot be compared directly [27,28]. Thus, only the behavior of individual trimethyl-substituted phosphonium IL compounds (e.g., [P1112O2]^+^, [P1115]^+^) as pure substances have been investigated so far [29]. Additionally, the stability of alkali metals towards such phosphonium IL derivatives is still unknown.

Imidazolium, pyrrolidinium and tetraalkylammonium-based ILs, for example, are currently widely studied for their suitability in Li-ion cells, especially in the case of metal anodes, and are typically used with [TFSI]^−^ or [FSI]^−^ as counter-ions [30,31,32,33]. Although imidazolium-based ILs show excellent results in terms of high conductivity (range of 1–10 mS∙cm^−1^ at 25 °C in Li-ion-based conducting salt mixtures) and low viscosity (range of 50–500 mPa∙s at 25 °C in Li-ion based conducting salt mixtures), their voltage stability is limited to the range < 4 V vs. Li/Li^+^ [34,35,36]. Pyrrolidinium- and tetraalkylammonium-based ILs exhibit improved voltage stability but are somewhat weaker in terms of ionic conductivity and Li mobility.

Due to their disadvantageous flow characteristics (comparably high viscosity), ILs are often used in mixtures with common carbonate-based electrolytes (e.g., IL + ethylene carbonate + dimethyl carbonate) [11,23,37,38,39], although in this case the desired properties in terms of safety improvement (flash point, flammability, etc. [19]) are just partially cancelled out by the low-boiling carbonates. On the other hand, the concentrations of conducting salt are often small in order to still maintain Li mobility, at least in part. However, this reduces the number of charge carriers in the electrolyte so that it can only handle lower current rates.

A major issue of many studies is their limitation to individual systems, e.g., one IL, which is then studied in several concentrations or mixing ratios. In this way, an estimation of similar systems or their behavior with other materials is difficult. This is, of course, is due to the fact that a comprehensive screening cannot always be carried out due to limited resources. Nevertheless, systematic investigations are important [9,13,40,41,42,43] in order to be able to better estimate a substance class as well as its application limits and behavior.

In this manuscript, n-alkyl-containing and ether-containing ionic liquids based on substituted trimethylphosphonium cations containing bis(fluorosulfonyl) imide or bis(trifluoromethanesulfonyl) imide anions are studied and are systematically compared. These ionic liquids were chosen as they contain comparably small cations with a sufficient degree of asymmetry to yield ionic liquids that are liquid at ambient temperature. A general finding for smaller ionic liquid cations is their increased dynamics leading to higher conductivities, which is desirable when building rechargeable batteries. The introduction of the ether group, which usually improves the characteristics of ionic liquids in multiple ways, allows us to compare the influence of this functionality on the general properties and stabilities of the ionic liquids. Modification of the side group length (butyl vs. pentyl and 2-ethoxy-ethyl vs. 2-methoxy-ethyl) allows us to monitor the effect of cation size on the electrolyte properties. In addition, we are able to compare the results with our previous investigations on ammonium ionic liquids [27], thus identifying the effect of changing the central atom from nitrogen to phosphorous. The anions bis(fluorosulfonyl)imide ([FSI]^−^) and bis(trifluoromethanesulfonyl)imide ([TFSI]^−^) are chosen as they are well known to yield ionic liquids with a beneficial property combination, including low solidification temperatures, fast dynamics and high stabilities [30,44,45]. For this purpose, the physicochemical properties, as well as selected electrochemical properties of the ILs with their corresponding Li salts, are carried out, and the obtained results are discussed. In this context, the aim of the work, besides showing comprehensive structure–property relationships, is to investigate the ranges of flow characteristics of phosphonium IL and phosphonium IL–salt mixtures and whether they are promising as sole solvents for battery applications. Their applicability in battery cells will be investigated in preliminary tests.

## 2. Results

### 2.1. Synthesis of the Ionic Liquids, Alkali Metal Compatibility and Thermal Properties

#### 2.1.1. Synthesis of the Phosphonium-Based Ionic Liquids and Electrolyte Mixtures

**Figure 1:**
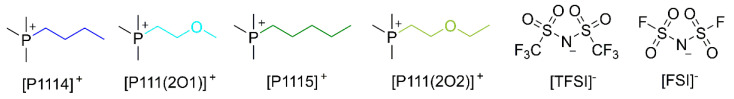
Molecular structures and abbreviation of the ionic liquid phosphonium cations and both anions, namely [TFSI]^−^ and [FSI]^−^. The numbers correspond to the number of C-atoms in the side chains (1: methyl; 4: butyl; 5: pentyl) or the alkoxy-substituent (2O1: 2-methoxy-ethyl; 2O2: 2-ethoxy-ethyl).

Within the scope of the study, ionic liquids with four different trimethyl phosphonium-based cations (Figure 1) were synthesized and analyzed. These cations were combined with two different anions, namely bis(fluorosulfonyl)imide ([FSI]^−^) and bis(trifluoromethanesulfonyl)imide ([TFSI]^−^), which are known to be highly stable in electrochemical applications and show favorable transport properties.

For the synthesis, the trimethyl phosphine precursor was quarternized with either alkyl bromide or the corresponding 2-alkoxy-ethyl ether bromide yielding the phosphonium bromides. The bromide salts were transformed into imide salts by metathesis with imide salts. Details of the syntheses and characterizations of the individual compounds are provided in the Supplementary Materials (chapter 1, SI).

The ionic liquids were mixed with conducting salt [Li][TFSI] and [Li][FSI] such that the anion of the IL matches the anion of the conducting salt to prepare binary IL-based electrolytes. In this way, uniform IL–salt mixtures were prepared so that interactions between different anions were excluded. An overview of all ionic liquids and binary ionic liquid salt mixtures is provided in Table 1. The molar mass *M*_e_ of the overall electrolyte is also provided, calculated according to Equation (1), including the density values below.

(1) $$M_{e}=\frac{m_{1}+m_{2}}{n_{1}+n_{2}}.$$

#### 2.1.2. Compatibility with Alkali Metals (Li, Na)

**Figure 2:**
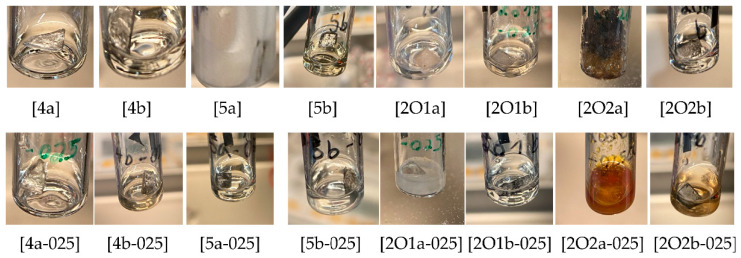
Images of the stored electrolytes (28 d) over lithium metal at 25 °C. a = [TFSI]^−^, b = [FSI]^−^.

**Figure 3:**
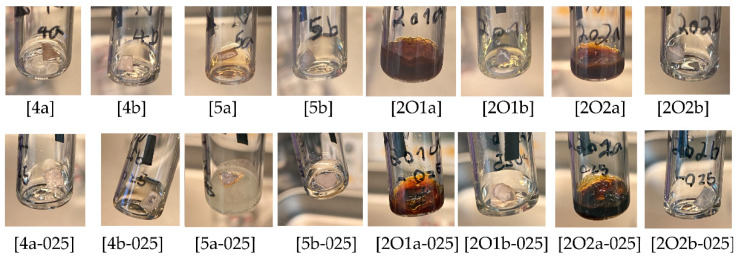
Images of the stored electrolytes (28 d) over sodium metal at 25 °C. a = [TFSI]^−^, b = [FSI]^−^.

Different ionic liquids and binary electrolytes were studied according to their compatibility and resilience against alkali metals. Often, such metals are used in electrochemical studies in order to evaluate the fundamental properties for applications, e.g., Li-ion or Na-ion-based batteries. For such tests, the compatibility against Li and Na is of particular interest due to the strong oxidative behavior of these metals, which are typically used as reducing agents. Both metals were tested at 25 °C and 70 °C due to reactivity and electrolyte color change within a period of two weeks, as summarized in Table 2. Typical photographs after the storage periods over lithium metal are shown in Figure 2 whereas the results for sodium storage are provided in Figure 3. It can be observed that pronounced reactions take place in the case of ether side chains of both ILs [P111(2O1)][TFSI] and [P111(2O2)][TFSI]. The IL with the ethoxyethyl side chain (2O2) is even more reactive compared to the methoxyethyl side chain (2O1) for Li, whereas no clear difference can be seen in the case of Na. However, both cations become stabilized when [FSI]^−^ is used as a counter anion instead of [TFSI]^−^. In contrast, the addition of conducting salt, namely 0.25 M [Li][TFSI] or 0.25 M [Li][FSI], results only in a slight deceleration of aging. Due to the relatively high melting point, the mixtures [5a/Li], [5a-025/Na] and [2O1a-025/Li] became solid while all others remained liquid even below their melting point (supercooled liquid). The results indicate that the reaction of alkoxyethyl side chains with Na is favored over that of alkyl side chains with Na, leading to degradation products, at least in the presence of [TFSI]^−^ ions. A more detailed explanation of this effect in terms of reactions potentially occurring is beyond the focus of this manuscript but is a starting point for future studies.

#### 2.1.3. Thermal Properties of the Ionic Liquids and Salt Mixtures

For most applications, the liquid state is an essential property to ensure sufficient ion mobility in the electrolyte; thus, a melting point below room temperature is of particular interest. To evaluate the phase transitions of the ionic liquids and IL salt mixtures, differential scanning calorimetry (DSC) measurements with slow scan rates were carried out, and the results are presented in Table 3. It is seen that the melting point of the pure phosphonium-based ionic liquids is relatively high, namely between 0–42 °C. When 0.25 M salt mixtures are prepared, the melting point as well as the crystallization temperature of the binary mixtures decrease slightly. The only IL in which a reduction of the melting point could not be detected was [2O2b]. Additionally, the ionic liquid [2O2a] with and without additional [Li][TFSI] salt was the only IL where ordered solids could not be observed. Due to the altered cation conformation of the ether side chains in the 2-ethoxy-ethyl derivatives, the effect of the additional conducting salt on the melting point might be limited [46,47]. Additionally, the influence and disturbance of additional ions in the IL matrix affect the well-known supercooling effect, which is more pronounced in salt mixtures. This effect results in a significant decrease in the crystallization temperatures when the absolute numbers are compared with the melting points (|Δ*T*_m_| = 2–6 °C vs. |Δ*T*_c_| = 2–17 °C).

#### 2.1.4. Density Values of Pure Ionic Liquids and Binary Salt Mixtures

**Figure 4:**
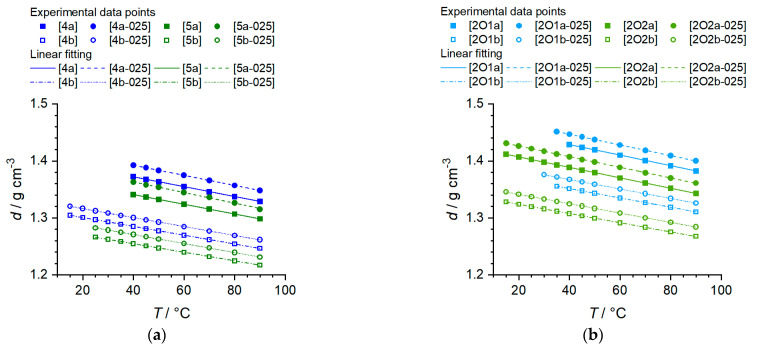
Density data of the ILs and IL–salt mixtures. (**a**) *n*-Alkyl phosphonium ionic liquids and salt mixtures; (**b**) Ether-containing phosphonium ionic liquids and IL salt mixtures.

The temperature-dependent density values were measured for all pure ILs and IL–salt mixtures. Since some compounds were solid at RT, as discussed before, the density values could only be measured at higher temperatures for such phosphonium IL or phosphonium IL salt mixtures. Figure 4 show the obtained values graphically. The experimental values, as well as the linear fitting data, are listed in Table S1a,b (Supplementary Materials).

It can be seen that the density values of all compounds and binary mixtures show a linear behavior over the entire temperature range. The values of the coefficient of determination of the linear regressions indicate a strictly linear dependence for all pure phosphonium ILs as well as phosphonium IL–salt mixtures (R^2^ > 0.9997). Moreover, it could be shown that the absolute values of the alkoxy-containing phosphonium ILs are higher than those of the alkyl-containing ILs. At the same time, lengthening of the side chain leads to consistently lower density values. The ILs with C4 side chains (4, 2O1) exhibit an approximate difference of Δ ≈ 0.03–0.04 g∙cm^−3^ compared to those with C5 side chains (5, 2O2). The addition of conducting salt ([Li][TFSI] or [Li][FSI]) further increases the corresponding values (Δ = 0.02 g∙cm^−3^). Similarly, the density values are decreased when [TFSI]^−^ is replaced by [FSI]^−^. This is plausible based on a molecular perspective. For example, compound 4a (d = 1.3720 g∙cm^−3^ at 40 °C) provides a molar volume *V*_m_ = (M∙n)/d of *V*_m,4a_ = 301 cm^3^∙mol^−1^, whereas 4b (d = 1.2848 g∙cm^−3^ at 40 °C) has a molar volume of only *V*_m,4b_ = 244 cm^3^∙mol^−1^. This is understandable as a result of the smaller molecular size of [FSI]^−^.

### 2.2. Transport Properties

#### 2.2.1. Viscosity of the Ionic Liquids and Ionic Liquid–Based Mixtures

**Figure 5:**
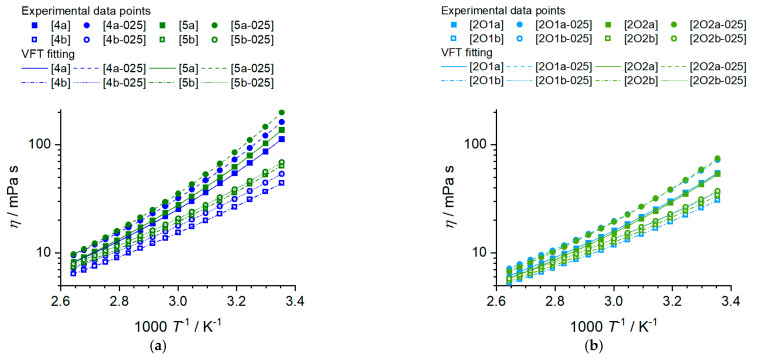
Dynamic viscosity in dependence of temperature for the bulk ILs and the IL–salt mixtures. (**a**) shows the *n*-alkyl phosphonium ionic liquids and salt mixtures and (**b**) the ether-containing phosphonium ionic liquids and IL salt mixtures.

The viscosity of a battery electrolyte is of central importance since it limits the transport of electroactive species and, therefore, the charging and discharging rates which can be applied to the batteries. For ionic liquids in battery applications, the viscosity is even more central, as the viscosity values are significantly higher than those of electrolytes based on molecular solvents and inversely proportional to the conductivity (see Walden relations Equation (2)). Finding structure–property relations to minimize the viscosity of ionic liquids is, therefore, a crucial aim for their successful implementation in rechargeable batteries. The viscosity values of the investigated phosphonium ionic liquid and their mixtures with lithium salts at 25 °C, the Vogel–Fulcher–Tammann (VFT) fitting parameters $\eta_{0}$, $B_{\eta }$ and $T_{0,\eta }$, the Angell strength parameter for the viscosity $\delta_{\eta }$ as well as the activation energy of the viscous flow $E_{a,\eta }$ are presented in Table 4. The temperature-dependent viscosity of the electrolytes is plotted in Figure 5. Experimental viscosity values are provided in the Supplementary Materials.

All ionic liquids and phosphonium IL salt mixtures incorporating the [FSI]^−^ anion show significantly lower viscosities than their [TFSI]^−^ counterparts with the same cation structure. For the alkylated samples, this effect is more pronounced as the viscosities of the [FSI]^−^ phosphonium ILs are less than half that of the heavier imides at 25 °C, while for the ether samples, the viscosity values are only 1.8 and 1.6 times higher for the [TFSI]^−^ anion compared to [FSI]^−^. The trend of lower viscosity for the bis(fluorosulfonyl)imide phosphonium IL remains at higher temperatures but the factor in which it differs from the bis(trifluoromethylsulfonyl)imide samples decreases. Dissolving lithium salts in ionic liquids increases viscosity in all cases. However, the factor for the viscosity increase is much larger for the electrolytes with [TFSI]^−^ anions. Again, increasing the temperature lowers the ratio of $\eta^{\mathrm{Li}\;\mathrm{solution}}$/$\eta^{\mathrm{IL}\;}$. Elongation of the side chain length from butyl to pentyl has only a minor influence on the viscosity compared to the incorporation of ether groups and the use of the bis(fluorosulfonyl)imide anion. Adding a methylene group in the side chain when an ether group is present has an even more minor influence than that observed for the hydrocarbon chain. The temperature-dependent viscosity of both the pure ionic liquids and their mixtures could be well fitted with the Vogel–Fulcher–Tammann equation, see Table 5 and Figure 5.

Since there are no crossovers in the order of the viscosity values with temperature, the same trend as found for the viscosity values is also found for the activation energy of the viscous flow, $E_{a,\eta }$. This means that larger $E_{a,\eta }$ values are found for samples with [TFSI]^−^, longer side chains and methylene instead of ether side groups. All values for $\delta_{\eta }$ and $E_{a,\eta }$ are similar to the isostructural ammonium ionic liquids, so replacing the central phosphorous by nitrogen has a comparably low influence, only with the general trend that the viscosity of phosphonium ionic liquids is slightly lower than that of ammonium ionic liquids [27].

#### 2.2.2. Ionic Conductivity

**Figure 6:**
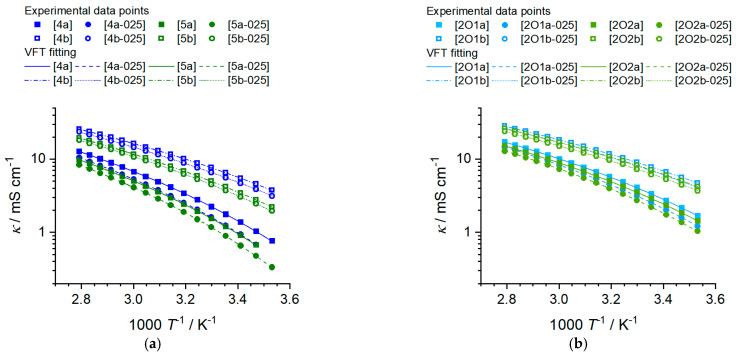
Conductivity data of the ILs and IL–salt mixtures. (**a**) *n*-alkyl phosphonium ionic liquids and salt mixtures; (**b**) ether-containing phosphonium ionic liquids and IL salt mixtures.

Conductivity is of central interest for the application of a particular electrolyte in electrochemical devices and applications. In the case of battery electrolytes, conductivity is a central factor limiting charge and discharge rates and affecting Ohmic losses and capacity of the battery. The electrolyte’s conductivity is thereby influenced by the mobility and amount of available charge carriers. Ionic liquids have a very high concentration of charge carriers as they are composed entirely of ions, but the strong, long-ranging Coloumbic interactions that are not shielded by solvent molecules, limit their ion mobility. Although ionic liquid-based electrolytes have advantages in terms of temperature range, electrochemical windows and safety, their conductivities are usually below those of conventionally used electrolytes based on molecular solvents. To mitigate these limitations, IL with high conductivities need to be found, which makes conductivity studies indispensable. The specific conductivity at 25 °C of the bulk ionic liquids and their binary mixture with lithium salts, the VFT-fitting parameters, $\kappa_{0}$, $B_{\kappa }$ and $T_{0,\kappa }$ Angell strength factor $\delta_{\kappa }$ and activation energy $E_{a,\kappa }$ (from 25 to 60 °C) for the specific conductivity are provided in Table 5. The temperature-dependent values are plotted in Figure 6. The experimental values are shown in the Supplementary Materials.

**Figure 7:**
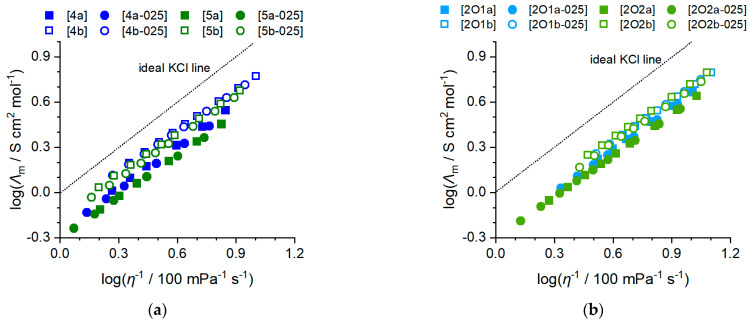
Walden plots of the electrolyte samples. (**a**) *n*-alkyl phosphonium ionic liquids and salt mixtures; (**b**) Ether-containing phosphonium ionic liquids and IL–salt mixtures. The dotted black line corresponds to the bisection, often termed the ‘ideal KCl line’ in the literature.

For specific conductivity, opposite trends compared to viscosity are found. This means, in particular, that the conductivity increases when the [FSI]^−^ anion is chosen, no lithium salt is added, the alkyl side chain is replaced by an ether chain and the chain length is reduced. As for viscosity, there is no crossover in κ values, so the order in which the conductivity increases is the same for all temperatures, see Figure 5. The values for Angell’s strength factor are all in a quite narrow range and adopt values from 3.32 to 4.58, which is a similar range than previously reported for ammonium ionic liquids with similar molecular structures [27]. The activation energies $E_{a,\kappa }$ of the specific conductivity are correlated to those of the viscous flow $E_{a,\eta }$ with the latter being systematically higher, but only to a small extent. The inverse trends in viscosity and conductivity of the bulk ionic liquids and their mixtures is to be expected, as the Walden relation 2 and 3, stating that the molar conductivity is inversely proportional to the viscosity, is well known to be valid for ILs [48,49].

(2) $$\Lambda_{m}\propto {\left(\eta^{-1}\right)}^{t}$$

(3) $$\log\Lambda_{m}=\log C+t\log\eta^{-1}$$

with *C* being a constant and t a fractional exponent close to unity. The resulting Walden plots of the phosphonium ionic liquids and mixtures with lithium salts are shown in Figure 7. Parameters for the fit according to the Walden relation are provided in the Supplementary Materials. All values of t lie in a small interval ranging from 0.88 to 0.92, which is to be expected due to the structural similarity and intermolecular interactions.

#### 2.2.3. Li Self-Diffusion Coefficients

The self-diffusion of lithium ions in the electrolytes is a measure of the mobility of the electroactive species and provides information about obtainable charging and discharging rates. Furthermore, information regarding the association and the liquid structure of the electroactive species can be obtained. The obtained lithium self-diffusion coefficients $D_{\mathrm{Li}}$ are provided in Table 6. The lithium self-diffusion coefficients show the same trends as previously observed for the conductivity. This means that for a common cation, the Li^+^ self-diffusion coefficients of the [FSI]^−^ ionic liquids are significantly higher than those with the [TFSI]^−^ anion. Elongation of the side chain leads to slower diffusion, with the effect being less significant for the [FSI]^−^ samples with ether side groups. For the ether containing [TFSI]^−^ mixtures, even a slight increase of the $D_{\mathrm{Li}}$ is observed. Using the Stokes–Einstein equation for stick conditions, Equation (4), it is possible to calculate the hydrodynamic radius $r_{h}$ of the lithium cation, see Table 6.

(4) $$D=\frac{k_{B}T}{6\pi \eta r_{h}}$$

with $k_{B}$ being Boltzmann’s constant. All obtained values for the hydrodynamic radius of lithium are approximately four to seven times higher than the radius of the lithium cation, indicating coordination of the lithium in the liquid state that hampers its diffusion. For the [TFSI]^−^ samples, the $r_{h}$ values are higher than for the [FSI]^−^ anions which indicates the coordination of anion A forming complexes of the type [Li(A)_x_]^1−^. These lithium–anion complexes are smaller in volume and radius for the [FSI]^−^ anion than for the [TFSI]^−^ anion, thus showing faster diffusion. The formation of such complexes of lithium with imide-type anions in bulk ionic liquids was also reported in the literature, for instance, using electrophoretic NMR [50,51] combined experiment and theory [52] as well as transport properties and IR spectroscopy [27,53].

### 2.3. Electrochemical Window and Aluminum Resistivity

In the following step, the electrochemical characteristics of the phosphonium ILs and phosphonium IL–salt mixtures were investigated. For this purpose, the voltage limits in which the electrolytes are oxidation-stable with respect to Al were determined, and basic lithium-ion-based cell tests were also carried out.

#### Cyclic Voltammetry Measurements, Potential Limit and Long-Term Potential Measurements

**Figure 8:**
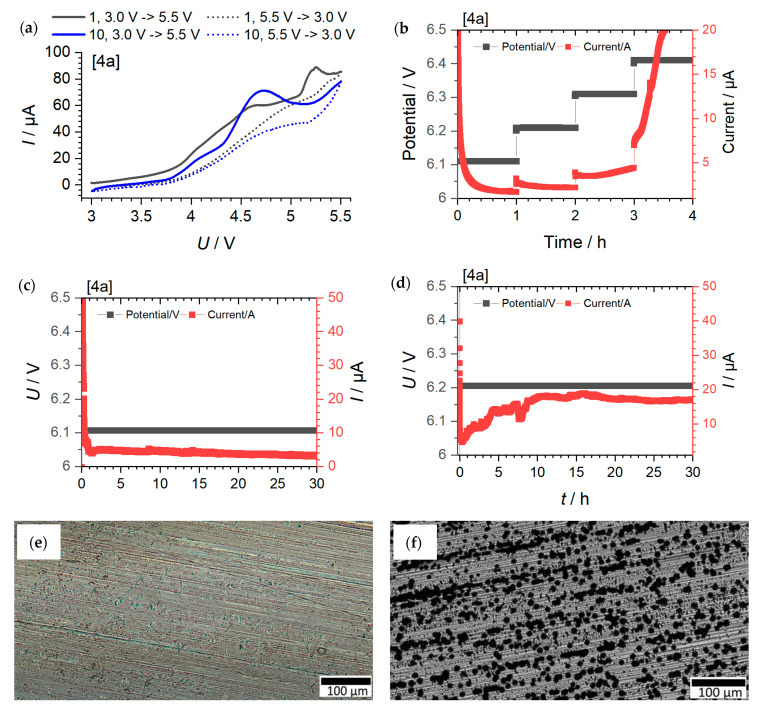
Aluminum corrosion tests exemplarily shown for electrolyte mixture [4a]. (**a**): Cyclic voltammetry (CV) measurement of Al//Li cells between 3.0–5.5 V vs. Li/Li^+^ of cycle 1 and cycle 10. (**b**): Polarization step method (Al//Li cells) with two descending current profiles (<6.3 V vs. Li/Li^+^) and two increasing current profiles (≥6.3 V vs. Li/Li^+^). (**c**): Long-term polarization at 6.1 V vs. Li/Li^+^ and decreasing current profile. (**d**): Long-term polarization of Al//Li cells at 6.2 V vs. Li/Li^+^ and increasing current profile. (**e**): Microscope image of long-term polarization at 6.1 V vs. Li/Li^+^ (cell 8c). (**f**): Microscope image of long-term polarization at 6.2 V vs. Li/Li^+^ (cell 8d) with visible corrosion pits.

The voltage stability with respect to aluminum was investigated using various electrochemical techniques. Figure 8 show the individual measurements as an example for compound [4a]. The determination of the potential limitations of the other ILs and IL–salt mixtures is shown in detail in the SI via chronopotentiometry measurements and related microscopy images. The results obtained from the individual measurements are listed in Table 7. In the first step, a conventional CV measurement was carried out in an Al/Li cell configuration (Figure 8a). An increase in current response is usually associated with an oxidation or decomposition process. In this case, a current increase is indicated at about 3.8 V vs. Li/Li^+^. This also persists in the further sequence of the CV measurement (after 10 cycles). Based on the CV method, this results in a voltage window of up to about 3.8 V vs. Li/Li^+^. However, there are different definitions up to which current value stability is provided. For example, a limit of 100 µA cm^−2^ is also commonly used. However, this value is not yet reached in the case investigated, even at 5.5 V vs. Li/Li^+^ (an electrode area of 1.13 cm^2^ results in a current density of about 71 µA cm^−2^). Consequently, such a definition suggests stability beyond 5.5 V vs. Li/Li^+^.

The next step was to investigate whether the current response behaves in a decreasing or increasing manner in the case of polarization measurements. An increasing behavior indicates oxidation or decomposition processes, e.g., Al corrosion. At the same time, this avoids exposing the system to significantly higher voltages so that decomposition products formed at higher voltages might have an influence on subsequent cycles (Figure 8b). In addition, processes associated with a one-time decomposition of electrolytes are partially hidden because, despite short-term decomposition, the overall current response decreases despite a possible short-time increase in current. It can be seen that during 1 h polarization (see also Figures S1–S8, Supplementary Materials), a decreasing current response is observed up to 6.2 V vs. Li/Li^+^, and an increasing current response is observed from 6.3 V vs. Li/Li^+^ or higher. On the basis of this measurement, stability up to 6.2 V vs. Li/Li^+^ can therefore be deduced. To verify this, long-term measurements (30 h) were carried out (Figure 8c,d). Here, it can be seen that in the case of polarization at 6.2 V, a current increase does occur again. At 6.1 V, on the other hand, the current drops continuously. Microscopy images of the two long-term tests at 6.1 and 6.2 V confirm the stability behavior that was found in the long-term polarization measurements. Consequently, the current stability was somewhat overestimated in the short-term measurement, whereas the long-term measurement confirms stability with respect to Al up to 6.1 V vs. Li/Li^+^.

Table 7 summarize the results of the individual measurements. It can be seen that both relaxation measurements (1h and 30 h polarization) provide similar potential limit values (oxidative stability), with the short-time measurement overestimating the voltage range to a certain extent. Pre-polarization at lower voltage may contribute to pretending too high stability values due to deposition processes which occur at lower potentials and increase the potential drop onto the interface layer at the surface of the Al sheet due to surface reactions (e.g., polymerization reactions) and surface deposition. In contrast, the CV measurements suggest oxidative stability values that are often considerably different from the polarization methods. Only in a few cases, nearly identical stability values are found (e.g., [2O1a], [2O2b], [5a-025]).

Based on the results of the long-term polarization measurements, where no signs of corrosion were visible in the microscopy examinations as well (see Figures S9–S16, Supplementary Materials), the following points can be concluded: Phosphonium ILs with ether side chains ([2O1], [2O2]) consistently show lower voltage stability compared to the alkyl-substituted phosphonium ILs ([4a], [5a]), regardless of the conducting salt. A possible explanation is the reactivity of the alkoxy group compared with the alkyl group, which leads to the higher reactivity of the corresponding compounds. The increase in chain length leads to significantly decreased voltage stability when there is no additional conducting salt, whereas, in the presence of additional conducting salt, the voltage stability is almost independent of the side chain length. In general, the effect of the presence of conducting salt does not indicate a typical trend; however, the [TFSI]-containing ILs exhibit a significantly higher overall voltage resistance or voltage stability compared to the [FSI]-containing ILs and IL-conducting salt mixtures. Based on the voltage stability of aluminum, the alkyl-substituted [TFSI]-based phosphonium ILs show good robustness to Al corrosion phenomena at >5.2 V vs. Li/Li^+^ even in the presence of [Li][TFSI], suggesting that they are expected to be stable even at correspondingly high cell voltages.

Overall, long-term measurement provides the best indication of oxidative stability with respect to Al, but the measurement effort here is very high. In this respect, short-term chronoamperometry measurement is a useful option for gaining a rough overview of the stability and then further refining the range in the long-term analysis.

### 2.4. Cell Test Results

#### 2.4.1. Proof of Principle in Coin Cell Tests

Cell tests were carried out with the phosphonium-based ionic liquids to prove the use in principle and to check the voltage stability. To avoid issues with graphite in terms of exfoliation and SEI formation, the materials lithium titanate oxide (LTO) and lithium iron phosphate (LFP) were used. This allowed the ILs to be tested directly without the need for the addition of additives that may also change basic electrolyte properties (e.g., stability limit, Li mobility, etc.). To allow comparability with the other measurement data, the concentration of 0.25 M was also used for the battery experiments. However, the consequence of this is that the number of charge carriers in the electrolyte is rather low, and only small currents are possible at acceptable internal resistances. For this reason, current rates between C/20 and C/50 were selected within the scope of the study.

**Figure 9:**
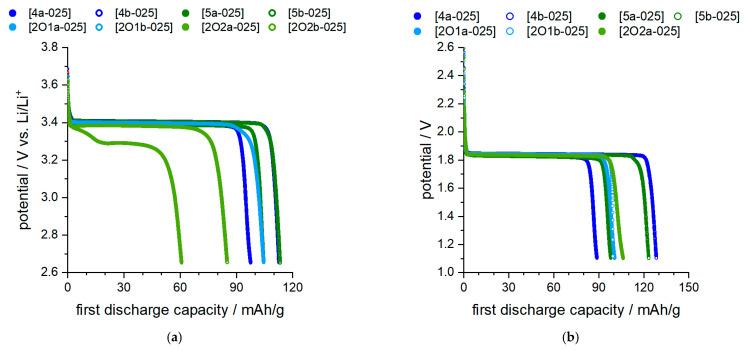
Cycle test in battery cells. (**a**): half cell, Li vs. LFP; (**b**): full cell, LTO vs. LFP.

Figure 9 illustrate the discharge curves of the LTO/LFP cells in the first cycle. Figure 9a show the half-cell tests of LFP vs. Li, and Figure 9b the full-cell tests of LFP vs. LTO. A visualization for better identification is shown in Figure S29 (half cells) and Figure S30 (full cells) in the SI for the TFSI and FSI-containing salts separately. All cells were repeated several times, and the individual measurements resulted in similar values. Despite the low C rate of C/50, it was not possible to extract a capacity of >110 mAh/g LFP in all cases. This indicates the limited mobility of ions both in the electrolyte and through the boundary layers and may also be associated with limited oxidative stability or the occurrence of corrosion phenomena. The half-cell measurements showed that the two ether-containing IL mixtures [2O2a-025] and [2O2b-025] lead to significant problems in metal contact (Li). The mixture [2O2b-025] could not be cycled in the full cell. Good or acceptable capacity values (>100 mAh/g) for half cells as well as full cell configuration were observed for mixtures [5b-025] and [4b-025].

**Figure 10:**
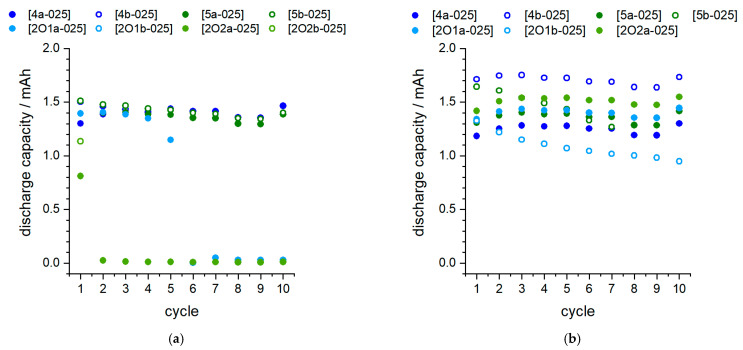
Cycle test in battery cells. (**a**): half-cell, Li vs. LFP; (**b**): full cell, LTO vs. LFP; current rates between 0.02–0.05 C.

**Figure 11:**
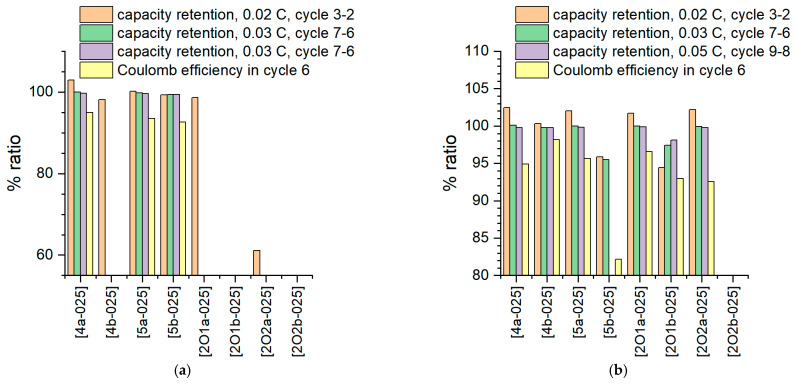
Cycle test in battery cells. (**a**): half cell, Li vs. LFP; (**b**): full cell, LTO vs. LFP; current rates as specified.

The cell test results are also confirmed with ongoing cycling (Figure 10). In the full cell configuration, IL mixtures [4b-025] and [2O2a-025] consistently demonstrate beneficial behavior within 10 cycles at a slow C rate, whereas in the half cell configuration, the electrolyte [2O2a-025] is not stable due to its reactivity with Li metal. Thus, sample [202a-025] shows a significantly lowered discharge plateau (Figure 9a). As noted above (Figure 2), exposure to Li without applied voltage already leads to a clear reaction between the electrolyte and Li. Despite several attempts, durable cycling was not successful in this case. It is therefore assumed that the surface reaction makes cycling impossible and leads to rapid degradation, which causes a lowered discharge step. This can also be seen in Figure 10a, where an immediate drop in discharge capacity occurs after the first cycle. The capacity retention is shown in more detail in Figure 11.

In half-cell arrangement, both mixtures [4a-025] and [5a-025] show good efficiency values, whereas in full cells without Li contact, mixtures [4b-025] and [2O1a-025] show promising behavior. All ether-containing IL mixtures could not be cycled against Li or could not be cycled in a stable manner. The low Coloumbic efficiency of mixture [2O2a-025] in the sixth cycle suggests that energy-consuming processes (e.g., ongoing decomposition) occur during cell charging, which have little effect on capacity aging but result in poor charge/discharge charge efficiency. In general, all IL mixtures exhibit low Coloumbic efficiency, significantly below 99%. Interestingly, compound [5b-025], which performed among the best in the first discharge step (both in the full cell and in the half cell), showed poor behavior during continued charging in the full cell, especially with respect to the Coloumbic efficiency.

#### 2.4.2. Overpotential Measurements in Half Cells and Full Cells

For a more detailed analysis of the potential differences between ether and alkyl-containing phosphonium ILs, the overpotential in LFP/LTO full cells was investigated in more detail. This refers to the difference in voltage plateaus between charging and discharging. The results are summarized in Table 8, and the details of the measurements are shown in Figure S31 (SI). It was observed that the alkyl-containing ILs tended to have higher overpotentials than the ether-containing ILs. The chain length showed no significant effect on the overpotential. There is a clear trend that [FSI]^−^-containing electrolytes lead to significantly lower overvoltages than [TFSI]^−^-containing ILs. This can be explained by the fact that the bulkier [TFSI]^−^ ions induce a stronger resistance to the newly approaching charge carriers (or the conducting salt ions) during accumulation in front of the interface than the significantly smaller [FSI]^−^ ions. The influence of the anions on the overpotential is greater than, for example, the direct comparison of the cations with different chain lengths so a significant influence of the anion can be assumed. Overall, the results show that the IL mixtures might be used as electrolytes in Li-ion cells. However, for an effective application, the conductive salt content would have to be increased, and a significant improvement of the cycling efficiencies (Coloumbic efficiency, overpotential effects) would have to be reached by additives.

Within the scope of the study, a brief comparison was made concerning the cycling capability between ammonium- and phosphonium-containing electrolytes. For this purpose, a saturated solution of lithium [bis(fluoro)](oxalato)borate (LiDFOB) in [N111(2O1)][TFSI] (approx. 0.6 mol∙kg^−1^) and [P111(2O1)][TFSI] (approx. 0.5 mol∙kg^−1^) was prepared (the solubility of LiDFOB in alkyl-substituted phosphonium and ammonium ILs is restricted). Half cells (NMC vs. lithium) were then constructed and cycled at 40 °C and 60 °C between C/50 and C/10, respectively (3–4.2 V vs. Li/Li^+^). The results are shown in Figure S32 (Supplementary Materials). It can be seen that at *T* = 40 °C, the performance continuously decreases to only 15–30% at C/10, which is a consequence of the flow characteristics in these ILs. In this case, the ammonium-based IL mixtures (~ 30% capacity retention) are superior to the phosphonium-based mixture (~15% capacity retention). However, it is observed that at *T* = 60 °C and C/20, a good cycling capability is achieved at a specific discharge capacity of 140–145 mAh∙g^−1^ in the case of the phosphonium-containing IL, whereas the ammonium-containing IL performs somewhat weaker at *T* = 60 °C and C/20 (115–118 mAh∙g^−1^). The results show that, in principle, stability up to 4.3 V vs. Li/Li^+^ is provided and that the phosphonium-containing electrolytes bring partial improvements compared to the ammonium-containing systems in such cell configurations.

## 3. Discussion

The phosphonium ILs were prepared according to known procedures from the literature and succeeded in high yields for the described substances of >80% over both steps of quaternization and salt exchange.

For density values, the same trends are found in the case of the phosphonium-containing ILs studied here as well as ammonium-containing ILs described earlier [27], so that also, in the case of the phosphonium ILs, a more compact steric arrangement of the alkoxy side chain (change in cation conformation when comparing alkoxy vs. alkyl side groups) is confirmed [46], which is accompanied by a decrease in molar volume and density values.

The lower viscosities of IL with [FSI]^−^ anion compared to samples with the [TFSI]^−^ anion and a common cation is a general finding [45] and can be rationalized with the smaller size and interaction with the cation of the former [52]. Literature reports state that the electrostatic and induced interaction energies of [FSI]^−^ anions with Li^+^ are lower than for the [TFSI]^−^ samples [52]. This could be used to explain the more marked increase in viscosity of the [TFSI]^−^ samples when adding the lithium salt as larger, more stable complexes of [Li(TFSI)_x_]^1−^ are formed that slow down the liquid dynamics [27]. Increasing the side chain length provides larger cations that resist the viscous flow and can induce a nanostructure in the ionic liquids consisting of nonpolar hydrocarbon domains and polar ionic domains. This formation of nanostructures is, for instance, reported for 1-alkyl-3-methyl imidazolium ionic liquids with alkyl chains longer or equally long to pentyl [54]; it increases the viscosity as a result of the higher liquid structuring [55,56]. The markedly lower viscosities of the ether-containing samples compared to the hydrocarbon analogues can be explained by altered cation conformation for the ether samples. While alkyl side chains are reported to prefer linear geometries, samples with 2-alkoxy-ethyl side chains often show curling of the side chain towards the positively charged cation center [46,47,57]. This altered cation conformation of the ether ILs causes more spherical cations and reduces the cation–anion interaction by shielding the cation charge and blocking coordination sites for the anion [47]. The curling of the ether side chain as a reason for the reduced ionic interactions also explains why the viscosity of the [2O2a] sample is slightly lower than for [2O1a], an effect also observed for ammonium-based ionic liquids [27]. Furthermore, the curling altering the cation conformation is also capable of explaining the differences in the densities when replacing a methylene group with ether oxygen. The Angell strength parameters for viscosity, $\delta_{\eta },$ decrease, thus the fragility of the liquids increases, upon replacing [FSI]^−^ by [TFSI]^−^, adding lithium salt, replacing alkyl by ether side chains and extending the side chains. These trends for the $\delta_{\eta }$ values of this phosphonium ionic liquids are also the same as found for similar ionic liquids with the ammonium cation paired with [FSI]^−^ and [TFSI]^−^ anions [27]. Overall the values obtain quite low values that are in the range commonly found for ionic liquids as highly fragile liquids [58].

In Walden analysis, exponents t smaller than unity which are found for the phosphonium ILs and phosphonium IL lithium salt mixtures here, are a common finding for ionic liquids [27,47,58,59] as molar conductivity and viscosity do not show an ideal reverse relationship, which also manifests in the slightly different activation energies for conductivity and viscosity [60]. All samples with the [FSI]^−^ anions are closer to the bisection of the Walden plot, which is sometimes referred to as the ‘ideal KC line’, although the assumption is somewhat arbitrary and has been criticized [48,61]. However, using the bisection of the Walden plot as a reference is commonly carried out in literature and can therefore serve to compare different classes of ionic liquids [2]. The distance to the bisection is then interpreted as a measure of ion association or ionicity. This would mean that the [FSI]^−^ ionic liquids have a lower degree of ion association which is beneficial for the use as electrolytes. With similar argumentation, the ionicity of the alkylated samples is higher than the ether substituted and is decreased upon side chain elongation, while adding lithium salt influences the ionicity only to a minor extent. Slightly lower viscosity values for ether-containing ionic liquids in comparison to their isostructural alkylated counterparts are also reported for other cation classes, such as phosphonium [40] or ammonium [27,47].

The electrochemical stability of the phosphonium ILs and phosphonium IL–salt mixtures towards aluminum was investigated by three different approaches, with the long-term polarization method showing the best matching with pitting phenomena appearing on the aluminum surfaces. Compared to the tetraalkyl phosphonium IL [(C_6_H_13_)_3_P(C_14_H_29_)][TFSI] described by Cha et al. which was investigated as a 1 M [Li][TFSI] electrolyte using CV measurements and which showed stability against Al up to about 4. 5 V vs. Li/Li^+^ [17], increased stability values of more than 5 V vs. Li/Li^+^ could be measured for the pure alkylphosphonium ILs (e.g., [4a], [5a]) in a mixture with [Li][TFSI] in this study. In contrast, the oxidative stability of [FSI]^−^-containing phosphonium ILs and [Li][FSI]-containing phosphonium IL–salt mixtures was significantly reduced with values of <4.5 V vs. Li/Li^+^. Similar to analogous ammonium-containing ILs, the stability decreased even further in some cases upon the addition of additional LiFSI [27]. Ether-containing phosphonium ILs consistently showed lower oxidative stability toward Al than the corresponding alkyl-based phosphonium ILs.

Using Li-ion cell tests, it was shown that cycling was possible in both half cells and full cells with the phosphonium IL mixtures, even at room temperature at low current rates. However, half-cell tests were only successful if there was sufficient chemical stability between lithium and the phosphonium IL mixture. Here, the alkyl-substituted phosphonium ILs showed significantly better stabilities than the ether-containing phosphonium ILs. Nevertheless, in the full-cell tests performed here (LFP vs. LTO), both mixtures [4b-025] and [2O1a-025] exhibited the most promising results. Overall, it was found that in spite of high viscosity values of >35 mPa∙s, cycling was possible even at room temperature, but blends with organic carbonates are expected to show improved cell performance due to better flow characteristics. Preliminary coin cell tests with NMC as a cathode material show promising results for phosphonium-based ILs compared to ammonium-based ILs.

## 4. Materials and Methods

### 4.1. Synthesis of the Ionic Liquids

The used phosphonium ionic liquids were synthesized by reaction of trimethyl phosphine with the corresponding 1-bromo alkyl or 1-bromo 2-alkoxy compounds to yield the bromide salts which were subjected to anion metathesis with the lithium imide salts. The phosphine was dissolved in dry, degassed acetonitrile under argon and 1.2 eq. of the 1-bromo compound were added. After stirring for 3 days at ambient temperature, the solvent and excess reagents were removed by rotary evaporation and dried on a Schlenk line. The phosphonium bromides were obtained in nearly quantitative yield as white solids.

For the anion metathesis, 1.0 eq of the bromide salts were dissolved in dry acetone, and 1.2 eq of [Li][FSI] or [Li][TFSI] were added subsequently. After stirring for 24 h, the mixture was filtered, the solvent was removed on a rotary evaporator, and the residue dissolved in dichloromethane and filtered. The organic phase was extracted four times with a slight amount of water, dried over MgSO_4_, filtered and the dichloromethane removed by rotary evaporation. In the case of the [FSI]^−^ ionic liquid, the aqueous washings of each extraction step were back extracted two times with dichloromethane and the organic phases combined. After removal of the solvent, the residues were dried for two days in a high vacuum with stirring. The identity and purity of the ionic liquids synthesized this way was checked by multinuclear NMR spectroscopy. The absence of halide residues was confirmed by testing with silver nitrate solution. The solutions of the phosphonium ionic liquids were prepared by dissolving a weighted lithium salt placed in a volumetric flask in the ionic liquid with the same anion. After complete dissolution of the lithium salt in the ionic liquid, the binary mixtures were transferred into a Schlenk flask and dried again in a high vacuum with stirring for two days. The dried samples were then handled using Schlenk techniques and a Labmaster 130 glove box (MBraun, Garching, Germany) to avoid contact with atmospheric moisture.

### 4.2. Thermal Transitions

The thermal behavior of the ionic liquids and binary mixtures was measured by differential scanning calorimetry (DSC) as reported in the literature [27]. Samples of approximately 10 mg ionic liquid or lithium solution were prepared in hermetically sealed aluminum crucibles in the glove box and measured on a DSC 1 STARe (Mettler Toledo, Gieβen, Germany) equipped with a liquid nitrogen cooling. The samples were heated from 25 °C to 120 °C using a heat rate of 5 °C min^−1^ to remove the thermal history. Afterwards, the samples were cooled to −120 °C with a cooling rate of −1 °C min^−1^ and subsequently reheated to 120 °C using a heating rate of +1 °C min^−1^. First order phase transitions (melting point, crystallization point) are provided as the maximum of the peak in the DSC traces; reported glass transitions were determined by the midpoint method.

### 4.3. Density

The density values of the ionic liquids and salt mixtures were obtained by a densitometer from Anton Paar (DMA4500M). All samples were measured in a temperature range between 15 °C and 90 °C as long as the samples were liquid. The standard uncertainty of the temperature during the measurement was u(T)=0.01 °C.

### 4.4. Viscosity

Temperature-dependent (dynamic) viscosities were measured on an MCR 301 rheometer (Anton Paar, Graz, Austria) which was placed on a vibration-isolated table. The cone-plate setup consisted of a CP50-1 cone (49.95 mm diameter and 1° cone angle) which was separated by a gap of 0.101 mm between cone tip and plate. All viscosity measurements were conducted under the flow of dry nitrogen to avoid the uptake of moisture from the atmosphere. The rheometer calibration was checked by measuring the temperature-dependent viscosity of a standard (Paragon Scientific, Prenton, UK) with given values (nominal viscosity of 129 mPa s at 25 °C). After temperature equilibration, the viscosity of the samples was measured for 30 values in the shear rate range from 50 to 150 s^−1^ with linear spacing, measuring each point for 15 s. As all viscosities at a particular temperature showed only Newtonian behavior, the shear-dependent viscosity values for each temperature were averaged. Values at other temperatures were measured in a similar way from 25 to 105 °C to construct the T-dependent viscosity curves. The temperature stability of the rheometer during the measurements was less than ± 0.01 °C.

### 4.5. Conductivity

Specific conductivities of the pure ionic liquid and binary solutions with lithium salts were obtained by impedance spectroscopy using a sealed commercial conductivity probe (WTW, Weilheim, Germany) consisting of two rectangular platinized platinum electrodes fused into glass with a nominal cell constant of 0.5 cm^−1^. The actual cell constant was determined prior to the experiments using commercial conductivity standards. The impedance spectra were obtained with an SP-150 potentiostat (Biologic, Seysinnet-Pariset, France) by applying voltages of 5, 10 and 15 mV and frequencies from 200 kHz to 1 Hz in 50 logarithmic steps. The electrolyte resistance for the three voltages was averaged. Temperature was controlled by immersing the conductivity probe in a Proline RP 1845 thermostat (LAUDA, Lauda-Königshofen, Germany). The specific conductivity κ was calculated as the actual cell constant divided by the electrolyte resistance R. From the specific conductivity and density, the molar conductivity was calculated using Equation (5), with *M* the molar mass of the sample.

(5) $$\Lambda_{M}=\frac{\kappa \;M}{\rho }.$$

### 4.6. Self-Diffusion Coefficients

^7^Li pulsed-field gradient self-diffusion coefficient measurements were carried out using a simulated echo-sequence (PFGSTE) as described elsewhere in detail [27]. Briefly, a Bruker NMR spectrometer serving at a ^1^H Larmor frequency of 300 MHz was used, and samples inside Hilgenberg tubes (glass no. 14, order no. 4007410) with an outer diameter of 1.0 mm, which were placed inside standard NMR tubes, where measured. All diffusion curves of the diffusing species i represented a single Gaussian function. The diffusion coefficient $D_{S}\left(Li\right)$ itself was determined from the Stejskal–Tanner Equation (6).

(6) $$I=I_{0}\cdot \exp\left[-D_{S}\left(Li\right)\cdot \gamma^{2}\cdot g^{2}\cdot \delta^{2}\cdot \left(\Delta -\frac{\delta }{3}\right)\right]$$

with I representing the intensity of the NMR signal for measurement with applied gradient,$\;I_{0}$ the initial signal intensity without applied gradient, γ the gyromagnetic ration (of the ^7^Li nucleus), g the applied gradient strength and δ the duration of the pulsed magnetic field gradient, as well as the diffusion time Δ.

### 4.7. Fitting of the Transport Properties

The temperature-dependent transport properties (κ, $\Lambda_{M}$, η) of pure ionic liquids and their lithium salt solutions could be fitted using the Vogel–Fulcher–Tammann(–Hesse) (VFT) Equation (7), which is commonly used to fit the transport properties of ionic liquids over a broad T-range [49].

(7) $$Y=Y_{0}\exp\left(\frac{B_{Y}}{T-T_{0,Y}}\right)$$

with Y being the fitted transport quantity, while $Y_{0}$, $B_{Y}$ and $T_{0,Y}$ (Vogel temperature) are material dependent parameters. Note that the parameter $B_{Y}$ obtains positive values for Y=η (η is decreasing with T) and negative values for the specific conductivity Y=κ and the molar conductivity $Y=\Lambda_{M}$ (conductivities are increasing with T). The Angell strength parameter $\delta_{Y}$ is the absolute value of $B_{Y}$ divided by $T_{0,Y}$ and used as a measure for liquid fragility. The parameter is also often termed D in the literature, which is not carried out here to avoid confusion with the diffusion coefficients. Liquids that are classified as ‘fragile’ have comparably small values of $\delta_{Y}$ and show faster changes in the transport properties than ‘strong liquids’ that have high strength parameters and nearly constant activation energy for the transport process. Angell’s strength parameter is directly linked to the kinetic fragility, which is an alternative measure for liquid fragility, by the relation $m=16+590\;\delta_{Y}{}^{-1}$[62]. Although the transport properties over a wide temperature range are better fitted with the VFT Equation (2), the Arrhenius Equation (8) is also commonly used to fit the transport properties over a narrow T-range with the benefit that it gives the vivid quantity of the activation energy which allows for a comparison to other liquids [63].

(8) $$\ln\left(Y\right)=\ln\left(Y_{0}\right)-\frac{E_{a,Y}}{RT}$$

with $E_{a,\;Y}$ being the activation energy of the transport quantity Y in the considered T-interval. Despite the positive viscosity values of $E_{a,\;Y}$, for conductivity, negative values were found.

### 4.8. Metal Compatibility

The extent to which the ILs and IL salt mixtures are stable in contact with Na and Li metal was investigated. For this purpose, 250 µL each of IL or IL salt mixture were placed in a vial, and a 0.5 × 0.5 cm piece of metal (Li, Na) was added to the glovebox. This was cut out of a larger, unrolled piece of metal. The sodium metal was previously cleaned of oil with pentane and dried carefully. The mixtures were kept sealed at room temperature inside the glovebox for a certain period of time.

### 4.9. Electrochemistry

Electrolyte handling was conducted in an argon-filled glove box (MBraun GmbH) with oxygen and water levels below 0.5 ppm. Polarization measurements were performed in the Al–Li electrode configuration. For this purpose, Swagelok cells were built with Al foil (d = 12 mm), Li foil (d = 12 mm), separator (QMA, d = 13 mm) and electrolyte (80 µL). At first, the rough potential stability range was determined by applying a defined voltage for 1 h, observing the current response, and then increasing the potential by 0.3 V each time the current response dropped. Based on the results, the voltage range was then further narrowed down to 0.1 V. Subsequently, long-term polarization was performed at the detected cut-off voltage for 20 h, and the current response was recorded and evaluated.

Cell tests were performed in LFP vs. LTO or LFP vs. Li metal configuration. For this purpose, LFP sheets (Custom Cells, 1 mAh∙cm^−2^, d = 16 mm) were built against LTO sheets (1 mAh∙cm^−2^, d = 16 mm) or lithium metal (Alfa Aesar, 99.9%, d = 15 mm, thickness = 750 µm) in coin cells (Hohsen) with 110 µL electrolyte and QMA (d = 17 mm) as separator. The cells were cycled at a C rate referenced to LFP (150 mAh∙g^−1^). The C-rate was set to: C/50 (3 cycles)–C/33 (4 cycles)–C/20 (2 cycles)–C/50 (1 cycle). NMC was used from Custom Cells with 2 mAh∙cm^−2^ and constructed in the same cell dimensions and configuration.

### 4.10. Cyclic Voltammetry

Cyclic voltammetry measurements were carried out at a Zahner X-Pot potentiostat. Briefly, three electrode cells (EL-Cell GmbH, Hamburg, Germany) were measured with Li (Ø = 17 mm) (reference/counter electrode), platinum (Ø = 18 mm) (working electrode) and a glass fiber separator (Whatman, QMA, Ø = 19 mm) including electrolyte (volume: 75 µL) in between. The potential range was set to 3–6.5 V vs. Li/Li^+^, and a scan speed of 1 mV∙s^−1^ was used.

### 4.11. Microscopy

An Olympus AX70 microscope was used for microscopy studies of the aluminum sheets after the corrosion tests. The aluminum foils were applied to glass plates to ensure they were flat-aligned. The microscope images were analyzed with the software Stream Desktop 2.5 from Olympus.

## 5. Conclusions

In summary, the study systematically investigated the structure–property relationships of alkyl- and ether-based phosphonium-containing ionic liquids (phosphonium ILs) with the two anions [TFSI]^−^ and [FSI]^−^. The study revealed that all phosphonium ILs, as well as their binary conducting salt mixtures ([Li][TFSI] and [Li][FSI]), exist in the liquid state above 40 °C and exhibit good stability towards alkali metals. Overall, the all-alkyl-based phosphonium ILs are significantly more stable against Li and Na metal than the analogous ether-containing phosphonium ILs. The ether-containing phosphonium ILs were found to have higher density values than the alkyl-containing ILs, while the addition of conducting salt further increased the density values. PFG-NMR analysis showed an aggregation of the Li ions towards Li coordination and Li complexes, respectively. The activation energies for viscosity and conductivity are highest in the case of alkyl-containing phosphonium ILs with [TFSI]^−^ as a counter-ion, and lowest in the case of ether-containing phosphonium ILs with [FSI]^−^ as a counter-ion. Overall, the pure ILs show low to moderate viscosity (η = 19–63 mPa∙s) and good to moderate flow properties, respectively. The transport properties increase in the case of (a) short side chains, (b) use of [FSI]^−^ instead of [TFSI]^−^, (c) use of ether-containing ILs and (d) without additional lithium conducting salt. The oxidative stability was evaluated using different techniques to find realistic cut-off potential limits of the different ILs and IL salt mixtures. The general usability as an electrolyte for Li-ion cells was confirmed in coin cell tests (half- as well as full-cell tests).

## Figures and Tables

**Table 1:** Overview of ionic liquids and electrolytes. The abbreviation was chosen for labeling the samples in the manuscript. “a” mentions the [TFSI]^−^ anion, and “b” is used when [FSI]^−^ salt is present.

Abbreviation	Ionic Liquid	Salt	Salt Concentration [mol L^−1^]	Molar Mass, *M*_e_ [g mol^−1^]
[4a]	[P1114][TFSI]	---	---	413.2
[4a-025]	[P1114][TFSI]	[Li][TFSI]	0.25	404.1
[4b]	[P1114][FSI]	---	---	313.2
[4b-025]	[P1114][FSI]	[Li][FSI]	0.25	305.8
[5a]	[P1115][TFSI^]^	---	---	427.2
[5a-025]	[P1115][TFSI]	[Li][TFSI^]^	0.25	416.5
[5b]	[P1115][FSI]	---	---	327.2
[5b-025]	[P1115][FSI]	[Li][FSI]	0.25	318.4
[2O1a]	[P111(2O1)][TFSI]	---	---	415.2
[2O1a-025]	[P111(2O1)][TFSI]	[Li][TFSI]	0.25	406.2
[2O1b]	[P111(2O1)][FSI]	---	---	315.2
[2O1b-025]	[P111(2O1)][FSI]	[Li][FSI]	0.25	308.0
[2O2a]	[P111(2O2)][TFSI]	---	---	429.2
[2O2a-025]	[P111(2O2)][TFSI]	[Li][TFSI]	0.25	418.6
[2O2b]	[P111(2O2)][FSI]	---	---	329.2
[2O2b-025]	[P111(2O2)][FSI]	[Li][FSI]	0.25	320.6

**Table 2:** Compatibility with alkali metals within two weeks (visible degradation). Following signs are used: “+”: metal remains stable without visible degradation; “c”: electrolyte with change of color; “−”: metal with surface modification and/or strong electrolyte reaction. Recognized changes are only visible modifications of the metal and/or liquid solution.

Ionic Liquid	Li [25 °C]	Li [70 °C]	Na [25 °C]	Na [70 °C]	Electrolyte Mixture	Li [25 °C]	Li [70 °C]	Na [25 °C]	Na [70 °C]
[4a]	+	+	−	−	[4a-025]	+	+	+	+
[4b]	+, c	+, c	−, c	−, c	[4b-025]	−, c	−, c	−, c	−, c
[5a]	+	+	−, c	−, c	[5a-025]	−, c	−, c	−, c	−, c
[5b]	−	−	+, c	+, c	[5b-025]	−, c	−, c	−, c	−, c
[2O1a]	+	+	−, c	−, c	[2O1a-025]	+	+	−, c	−, c
[2O1b]	−	−	−, c	−, c	[2O1b-025]	+	+, c	−	−
[2O2a]	−	−	−, c	−, c	[2O2a-025]	−, c	−, c	−, c	−, c
[2O2b]	−	−	+	+	[2O2b-025]	−, c	−	−	−

**Table 3:** Thermal properties of the ionic liquids and ionic liquid binary salt mixtures obtained by DSC measurements at 1 K∙min^−1^ scanning rate, including the melting point (*T*_m_), glass transition (*T*_g_) and crystallization temperature (*T*_c_). The uncertainty in the temperature is ±1 °C.

Ionic Liquid	*T*_g_/°C	*T*_c_/°C	*T*_m_/°C	Electrolyte Mixture	*T*_g_/°C	*T*_c_/°C	*T*_m_/°C
[4a]	-	−12	42	[4a-025]	-	−14	39
[4b]	-	−36	0	[4b-025]	-	−53	−2
[5a]	-	−9	41	[5a-025]	−70	-	39
[5b]	-	−5	28	[5b-025]	-	−13	24
[2O1a]	-	−20	42	[2O1a-025]	-	−36	36
[2O1b]	-	−3	36	[2O1b-025]	-	−7	33
[2O2a]	−82	-	-	[2O2a-025]	−78	-	-
[2O2b]	-	-38	8	[2O2b-025]	-	−47	9

**Table 4:** Viscosity η at 25 °C, VFT fitting parameters ($\eta_{0}$,$\;B_{\eta }$, $T_{0,\eta }$), Angell strength factor $\delta_{\eta }$ and activation energy $E_{a,\eta }$ (in the range of 25 to 60 °C) for the T -dependent viscosity data, following Equation (7).

Sample	*η*^25 °C^ mPa∙s	*η*_0_/10^−1^ mPa∙s	*B_η_*/K	*T*_0,_*_η_*/K	R^2^	$\delta_{\eta }=B\;/T_{0}$	*E*_a,__η_ /kJ∙mol^−1^
[4a]	112.3	1.66 ± 0.05	772.8 ± 8.8	179.6 ± 0.8	>0.99999	4.30	35.1 ± 0.6
[4a-025]	162.0	1.67 ± 0.05	783.2 ± 7.5	184.3 ± 0.6	>0.99999	4.25	38.2 ± 0.7
[4b]	44.19	1.89 ± 0.05	796.4 ± 8.3	152.1 ± 0.9	>0.99999	5.24	24.8 ± 0.3
[4b-025]	53.77	2.33 ± 0.04	747.6 ± 4.9	160.8 ± 0.5	>0.99999	4.65	26.1 ± 0.4
[5a]	136.7	1.34 ± 0.01	812.2 ± 2.5	181.0 ± 6.9	>0.99999	4.49	37.5 ± 0.7
[5a-025]	198.7	1.29 ± 0.02	837.2 ± 3.9	184.0 ± 0.9	>0.99999	4.55	38.0 ± 2.1
[5b]	63.27	1.43 ± 0.32	887.3 ± 54.1	152.8 ± 6.9	0.99999	5.81	28.2 ± 0.3
[5b-025]	68.86	2.19 ± 0.06	765.9 ± 9.0	164.9 ± 0.9	>0.99999	4.64	28.1 ± 0.4
[2O1a]	54.79	2.06 ± 0.03	694.0 ± 4.1	173.9 ± 0.4	>0.99999	3.99	28.9 ± 0.5
[2O1a-025]	72.05	2.00 ± 0.06	727.1 ± 9.2	174.6 ± 0.9	0.99999	4.16	30.7 ± 0.5
[2O1b]	30.66	1.92 ± 0.04	765.1 ± 8.2	147.3 ± 0.9	>0.99999	5.20	22.5 ± 0.3
[2O1b-025]	36.46	2.38 ± 0.05	714.5 ± 6.8	156.2 ± 0.8	>0.99999	4.57	23.5 ± 0.3
[2O2a]	53.34	1.91 ± 0.03	694.4 ± 3.9	174.8 ± 0.4	>0.99999	3.97	29.3 ± 0.5
[2O2a-025]	74.42	1.65 ±0.02	756.4 ± 2.9	174.4 ± 0.3	>0.99999	4.34	31.7 ± 0.5
[2O2b]	33.83	1.96 ± 0.03	757.7 ± 4.8	151.0 ± 0.5	>0.99999	5.02	23.3 ± 0.3
[2O2b-025]	37.26	2.15 ± 0.07	737.3 ± 11.0	155.1 ± 1.2	0.99999	4.75	23.9 ± 0.3

**Table 5:** Specific conductivity κ at 25 °C, VFT fitting parameters ($\kappa_{0}$,$\;B_{\kappa }$, $T_{0,\kappa }$ ) Angell strength parameter $\delta_{\kappa }$ and activation energy $E_{a,\kappa }$ (in the range of 25 to 60 °C) for the T -dependent conductivity data.

Sample	*κ^25°^*^C^/ mS∙cm^−1^	*κ*_,0_ / mS∙cm^−1^	*B_κ_* /K	*T*_0_,*κ*/K	R^2^	$\delta_{\kappa }=\left|B_{0}/T_{0,\kappa }\right|\;$	*E*_a,__κ_ /kJ∙mol^−1^
[4a]	1.76	600.5 ± 13.1	−684.6 ± 6.5	180.7 ± 0.7	>0.99999	3.79	31.6 ± 0.6
[4a-025]	1.24	632.1 ± 26.2	−719.2 ± 12.4	182.8 ± 1.3	0.99998	3.93	34.2 ± 0.6
[4b]	6.49	582.3 ± 8.0	−615.3 ± 4.6	161.3 ± 0.6	>0.99999	3.82	21.6 ± 0.3
[4b-025]	5.57	767.4 ± 36.8	−717.3 ± 16.8	152.5 ± 2.0	0.99998	4.70	22.5 ± 0.3
[5a]	1.20	536.9 ±15.4	−698.6 ± 8.5	183.7 ± 0.9	>0.99999	3.80	33.7 ± 0.6
[5a-025]	0.89	528.7 ± 18.2	−711.3 ± 10.0	186.8 ± 1.0	0.99999	3.81	36.0 ± 0.6
[5b]	4.16	607.9 ± 7.8	−669.5 ± 4.2	163.9 ± 0.5	>0.99999	4.09	24.3 ± 0.3
[5b-025]	3.73	537.2 ± 15.6	−640.1 ± 9.2	169.4 ± 1.1	0.99999	3.78	25.0 ± 0.3
[2O1a]	3.35	506.2 ± 20.0	−626.3 ± 12.2	173.4 ± 1.5	0.99998	3.61	25.7 ± 0.4
[2O1a-025]	2.53	559.5 ± 47.2	−672.9 ± 26.3	173.4 ± 3.0	0.99994	3.88	27.8 ± 0.6
[2O1b]	7.79	665.1 ± 32.8	−656.4 ± 17.3	150.6 ± 2.3	0.99997	4.36	20.1 ± 0.3
[2O1b-025]	6.94	563.4 ± 8.2	−609.7 ± 4.8	159.5 ± 0.7	>0.99999	3.82	20.9 ± 0.3
[2O2a]	2.89	446.3 ± 5.9	−618.2 ± 4.0	175.5 ± 0.5	>0.99999	3.52	26.3 ± 0.4
[2O2a-025]	2.20	396.1 ± 23.2	−603.7 ± 17.8	181.8 ± 2.1	0.99998	3.32	28.2 ± 0.5
[2O2b]	7.07	555.2 ± 12.1	−615.0 ± 7.4	157.2 ± 1.0	0.99999	3.91	20.4 ± 0.3
[2O2b-025]	6.14	641.5 ± 76.1	−686.3 ± 41.9	149.9 ± 5.3	0.99986	4.58	20.9 ± 0.5

**Table 6:** Lithium self-diffusion coefficients for conducting salt-containing binary mixtures at 298 K.

Electrolyte	*D*_Li_/10^−12^ m^2^ s^−1^	$r_{h}$ $/10^{-10}\;m$	Electrolyte	*D*_Li_/10^−12^ m^2^ s^−1^	$r_{h}$ $/10^{-10}\;m\;$
[4a-025]	3.7 ± 0.1	3.64 ± 0.12	[2O1a-025]	6.9 ± 1.2	4.39 ± 0.77
[4b-025]	19.0 ± 1.0	2.14 ± 0.12	[2O1b-025]	26.0 ± 0.7	2.30 ± 0.08
[5a-025]	2.8 ± 0.2	3.93 ± 0.29	[2O2a-025]	8.2 ± 0.3	3.58 ± 0.15
[5b-025]	14.0 ± 0.3	2.27 ± 0.07	[2O2b-025]	24.0 ± 0.4	2.44 ± 0.06

**Table 7:** Potential limits in Al vs. Li Swagelok-based cells which are received from (1) CV measurements, (2) potential step sequence measurements (*U*_ramping_, polarization for 1 h) and (3) 30 h polarization tests with overall decreasing current density (*U*_max,pol_, polarization for 30 h).

Ionic Liquid	Potential Limit from CV/V	Potential Limit via U-Ramping/V	Potential Limit Confirmed at 30 h Polarization	Electrolyte Mixture	Potential Limit from CV/V	Potential Limit via U-Ramping/V	Potential Limit Confirmed at 30 h Polarization
[4a]	3.8	6.2	6.1	[4a-025]	>5.5	5.3	5.2
[4b]	3.9	4.1	4.4	[4b-025]	4.0	4.4	4.4
[5a]	5.3	5.8	5.5	[5a-025]	5.3	5.3	5.3
[5b]	3.9	4.3	4.5	[5b-025]	4.2	3.7	3.7
[2O1a]	4.8	4.7	4.6	[2O1a-025]	>5.5	5.0	4.8
[2O1b]	5.2	3.3	3.2	[2O1b-025]	4.2	3.4	3.4
[2O2a]	3.5	2.4	2.1	[2O2a-025]	3.5	5.0	4.8
[2O2b]	3.3	3.3	3.1	[2O2b-025]	3.8	3.3	3.1

**Table 8:** Overpotential of charging vs. discharging plateau during the first cycle at C/50 in LFP/LTO cells at room temperature (*T* = 298 °C).

Electrolyte Mixture	Δ*U*/mV	Electrolyte Mixture	Δ*U*/mV
[4a-025]	78	[2O1a-025]	60
[4b-025]	52	[2O1b-025]	50
[5a-025]	82	[2O2a-025]	63
[5b-025]	56	[2O2b-025]	---

## Data Availability

Not applicable.

## Supplementary Materials

The following are available online at https://www.mdpi.com/article/10.3390/molecules27154729/s1, Table S1a: Density values given in g mL^−1^ of the ILs and IL–salt mixtures. Table S1b: Linear fitting values for density. Table S2: Viscosity values η in mPa s of the ILs and IL–salt mixtures. Table S3: Specific conductivity values κ in mS cm^−1^ of the ILs and IL–salt mixtures. Table S4: Molar conductivity values of the ILs and IL–salt mixtures. Table S5: Fitting parameters for the Walden relation. Figure S1: Polarization ramping to obtain the potential limit U_ramping_ in V for [4a] (a) and [4a-025]. Figure S2: Polarization ramping to obtain the potential limit U_ramping_ in V for [5a] (a) and [5a-025]. Figure S3: Polarization ramping to obtain the potential limit U_ramping_ in V for [201a] (a) and [201a-025]. Figure S4: Polarization ramping to obtain the potential limit U_ramping_ in V for [202a] (a) and [202a-025]. Figure S5: Polarization ramping to obtain the potential limit U_ramping_ in V for [4b] (a) and [4b-025]. Figure S6: Polarization ramping to obtain the potential limit U_ramping_ in V for [5b] (a) and [5b-025]. Figure S7: Polarization ramping to obtain the potential limit U_ramping_ in V for [201b] (a) and [201b-025]. Figure S8: Polarization ramping to obtain the potential limit U_ramping_ in V for [202b] (a) and [202b-025]. Figure S9: Microscopy images of the samples [4a] (a) and [4a-025] (b) after 30 h polarization at U_max, pol_. Figure S10: Microscopy images of the samples [5a] (a) and [5a-025] (b) after 30 h polarization at U_max, pol_. Figure S11: Microscopy images of the samples [201a] (a) and [201a-025] (b) after 30 h polarization at U_max, pol_. Figure S12: Microscopy images of the samples [202a] (a) and [202a-025] (b) after 30 h polarization at U_max, pol_. Figure S13: Microscopy images of the samples [4b] (a) and [4b-025] (b) after 30 h polarization at U_max, pol_. Figure S14: Microscopy images of the samples [5b] (a) and [5b-025] (b) after 30 h polarization at U_max, pol_. Figure S15: Microscopy images of the samples [201b] (a) and [201b-025] (b) after 30 h polarization at U_max, pol_. Figure S16: Microscopy images of the samples [202b] (a) and [202b-025] (b) after 30 h polarization at U_max, pol_. Figure S17: Linear voltammetry scan of Al vs. Li cells from 3.0 to 5.5 V vs. Li/Li^+^ of (a) n-alkyl phosphonium ionic liquids and (b) n-alkyl phosphonium ionic liquid salt mixtures. Figure S18: Linear voltammetry scan of Al vs. Li cells from 3.0 to 5.5 V vs. Li/Li^+^ of (a) ether containing phosphonium ionic liquids and (b) ether containing phosphonium ionic liquid salt mixtures. Figure S19: Microscopy images of the aluminum sheets including alcyl phosphonium IL and IL/salt electrolytes after the CV experiment. Figure S20: Microscopy images of the aluminum sheets including ether containing phosphonium IL and IL/salt electrolytes after the CV experiment. Figure S21: Cyclic voltammetry (CV) measurement of Al//Li cells between 3.0–5.5 V vs. Li/Li^+^ of cycle 1 and cycle 10 of electrolyte [4a] (a) and electrolyte [4a_025] (b). Figure S22: Cyclic voltammetry (CV) measurement of Al//Li cells between 3.0–5.5 V vs. Li/Li^+^ of cycle 1 and cycle 10 of electrolyte [5a] (a) and electrolyte [5a_025] (b). Figure S23: Cyclic voltammetry (CV) measurement of Al//Li cells between 3.0–5.5 V vs. Li/Li^+^ of cycle 1 and cycle 10 of electrolyte [2O1a] (a) and electrolyte [2O1a_025] (b). Figure S24: Cyclic voltammetry (CV) measurement of Al//Li cells between 3.0–5.5 V vs. Li/Li^+^ of cycle 1 and cycle 10 of electrolyte [2O2a] (a) and electrolyte [2O2a_025] (b). Figure S25: Cyclic voltammetry (CV) measurement of Al//Li cells between 3.0–5.5 V vs. Li/Li^+^ of cycle 1 and cycle 10 of electrolyte [4b] (a) and electrolyte [4b_025] (b). Figure S26: Cyclic voltammetry (CV) measurement of Al//Li cells between 3.0–5.5 V vs. Li/Li^+^ of cycle 1 and cycle 10 of electrolyte [5b] (a) and electrolyte [5b_025] (b). Figure S27: Cyclic voltammetry (CV) measurement of Al//Li cells between 3.0–5.5 V vs. Li/Li^+^ of cycle 1 and cycle 10 of electrolyte [2O1b] (a) and electrolyte [2O1b_025] (b). Figure S28: Cyclic voltammetry (CV) measurement of Al//Li cells between 3.0–5.5 V vs. Li/Li^+^ of cycle 1 and cycle 10 of electrolyte [2O2b] (a) and electrolyte [2O2b_025] (b). Figure S29. Cycle test in half cell configuration. Figure S30: Cycle test in full cell configuration. Figure S31: dQ/dU during the first cycle for measuring the overpotential. Figure S32: Comparison of ammonium and phosphonium-based electrolytes in NMC vs. Li cell configuration.

## Sample Availability

Not available.
