# Supplementary material for: Novel Phosphonium-Based Ionic Liquid Electrolytes for Battery Applications
Source: Molecules. 2022 Jul 24;27(15):4729. doi: 10.3390/molecules27154729 (PMC9329924; doi:10.3390/molecules27154729)
Supplement: Supplementary file 1 [file molecules-27-04729-s001.zip › molecules-1822308-supplementary.pdf]

Supplementary data to:

Novel Phosphonium-Based Ionic Liquid Electrolytes for Battery Applications

Andreas Hofmann [1,*], Daniel Rauber [2], Tzu-Ming Wang [1], Rolf Hempelmann [2], Christopher W. M. Kay [2,3] and Thomas Hanemann [1,4]

1 Institute for Applied Materials, Karlsruhe Institute of Technology, Hermann-von-Helmholtz-Platz 1,
D-76344 Eggenstein-Leopoldshafen, Germany; uvdyf@student.kit.edu (T.-M.W.); thomas.hanemann@kit.edu (T.H.)

2 Department of Chemistry, Saarland University, Campus B2.2, 66123 Saarbrücken, Germany;
daniel.rauber@uni-saarland.de (D.R.); r.hempelmann@mx.uni-saarland.de (R.H.); christopher.kay@uni-saarland.de (C.W.M.K.)

3 London Centre for Nanotechnology, Bloomsbury Campus, University College London, 17-19 Gordon Street,
London WC1H 0AH, UK

4 Department of Microsystems Engineering, University of Freiburg, Georges-Köhler-Allee 102,
D-79110 Freiburg, Germany

* Correspondence: andreas.hofmann2@kit.edu; Tel.: +49-721-608-25920

1. Synthesis of the Ionic Liquids

1.1 Synthesis of the halide precursors

Phosphonium bromide salts were synthesized from 1.0 eq. trimethylphosphine and 1.2 eq. to the alkyl-bromide in dry, degassed acetonitrile under argon atmosphere. After stirring for 3 days at ambient temperature, the solvent and excess reagents were removed by rotary evaporation and the residue dried in a high vacuum to obtain the bromide salts as colorless, hygroscopic solids in nearly quantitative yields.

1.1.1 Butyltrimethylphosphonium bromide [P1114][Br]

^{1}H-NMR (400 MHz; DMSO-d6): *δ*/ppm = 2.29 – 2.17 (m, 2H, P-CH_2), 1.86 (d, *J* = 14.9 Hz, 9H, P-CH_3), 1.55 – 1.43 (m, 2H, P-CH_2-$\boldsymbol{CH_2}$), 1.42 – 1.31 (m, 2H, $\boldsymbol{CH_2}$-CH_3), 0.90 (t, *J* = 7.2 Hz, 3H, CH_2-$\boldsymbol{CH_3}$).

^{13}C{^{1}H}-NMR (101 MHz; DMSO-d6): *δ*/ppm = 23.16 (d, *J* = 16.5 Hz, $\boldsymbol{CH_2}$-CH_3), 23.53 (d, *J* = 4.3 Hz, P-CH_2-$\boldsymbol{CH_2}$), 21.89 (d, *J* = 52.3 Hz, P-CH_2), 13.26 (s, CH_2-$\boldsymbol{CH_3}$), 7.21 (d, *J* = 53.8 Hz, P-CH_3).

^{31}P{^{1}H}-NMR (162 MHz; DMSO-d6): *δ*/ppm = 28.11 (s).

1.1.2 Trimethylpentylphosphonium bromide [P1115][Br]

^{1}H-NMR (400 MHz; DMSO-d6): *δ*/ppm = 2.19 – 2.07 (m, 2H, P-CH_2), 1.81 (d, *J* = 14.6 Hz, 9H, P-CH_3), 1.56 – 1.44 (m, 2H, P-CH_2-$\boldsymbol{CH_2}$), 1.42 – 1.25 (m, 4H, P-CH_2-$\boldsymbol{CH_2}$ + $\boldsymbol{CH_2}$-CH_3), 0.89 (t, *J* = 7.0 Hz, 3H, CH_2-$\boldsymbol{CH_3}$).

^{13}C{^{1}H}-NMR (101 MHz; DMSO-d6): *δ*/ppm = 32.23 (d, *J* = 16.0 Hz, P-$(CH_2)_2$-$\boldsymbol{CH_2}$), 22.19 (d, *J* = 52.9 Hz, P-CH_2), 21.35 (s, $\boldsymbol{CH_2}$-CH_3) 20.27(d, *J* = 4.3 Hz, P-CH_2-$\boldsymbol{CH_2}$), 13.59 (s, CH_2-$\boldsymbol{CH_3}$), 7.13 (d, *J* = 54.3 Hz, P-CH_3).

^{31}P{^{1}H}-NMR (162 MHz; DMSO-d6): *δ*/ppm = 28.84 (s).

1.1.3 (2-methoxyethyl)trimethylphosphonium bromide [P1112O1][Br]

^{1}H-NMR (400 MHz; DMSO-d6): *δ*/ppm = 3.66 (dt, *J* = 19.4 Hz, *J* = 6.4 Hz, 2H, P-CH_2-$\boldsymbol{CH_2}$), 3.27 (s, 3H, O-CH_3), 2.62 (dt, *J* = 13.9 Hz, *J* = 6.2 Hz, 2H, P- CH_2) 1.91 (t, *J* = 15.2 Hz, 9H, P-CH_3).

^{13}C{^{1}H}-NMR (101 MHz; DMSO-d6): *δ*/ppm = 64.81 (d, *J* = 6.0 Hz, P-CH_2-$\boldsymbol{CH_2}$), 58.03 (s, O-CH_3), 23.28 (d, *J* = 52.6 Hz, P-CH_2), 8.12 (d, *J* = 54.0 Hz, P-CH_3).

^{31}P{^{1}H}-NMR (162 MHz; DMSO-d6): *δ*/ppm = 27.76 (s).

1.1.4 (2-ethoxyethyl)trimethylphosphonium bromide [P1112O2][Br]

^{1}H-NMR (400 MHz; DMSO-d6): *δ*/ppm = 3.74 (dt, *J* = 19.9 Hz, *J* = 6.1 Hz, 2H, P-CH_2-$\boldsymbol{CH_2}$), 3.48 (q, *J* = 7.0 Hz, O-$\boldsymbol{CH_2}$-CH_3), 3.36 (s, 3H, CH_2-$\boldsymbol{CH_3}$), 2.54 – 2.46 (m, 2H, P-CH_2), 1.83 (t, *J* = 15.0 Hz, 9H, P-CH_3), 1.14 (t, *J* = 7.0 Hz, 3H, CH_2-$\boldsymbol{CH_3}$).

^{13}C{^{1}H}-NMR (101 MHz; DMSO-d6): *δ*/ppm = 65.73(s, $\boldsymbol{CH_2}$-CH_3) 62.81 (d, *J* = 6.5 Hz, P-CH_2-$\boldsymbol{CH_2}$), 23.67 (d, *J* = 53.9 Hz, P-CH_2), 14.91 (s, CH_2-$\boldsymbol{CH_3}$), 8.12 (d, *J* = 54.1 Hz, P-CH_3).

^{31}P{^{1}H}-NMR (162 MHz; DMSO-d6): *δ*/ppm = 26.82 (s).

2.1 Synthesis of the phosphonium bis(trifluoromethanesulfonyl)imde ionic liquids

Bis(trifluoromethanesulfonyl)imide ionic liquids were prepared by dissolving 1.0 eq. phosphonium bromide salt in dry acetone and adding 1.2 eq. [Li][NTf_2] salt. After stirring for 24 hours, the mixtures were filtered, and the acetone was removed by rotary evaporation. The residue was dissolved in dichloromethane and filtered again. The dichloromethane phase was extracted with a slight amount of water four times. The organic phase was dried over $MgSO_4$, and the solvent was removed by rotary evaporation. The residue was dried in a high vacuum for two days with stirring, yielding the ionic liquids in nearly quantitative yield.

1.2.1 Butyltrimethylphosphonium bis(trifluoromethanesulfonyl)imide [P1114][TFSI]/[4a]

^{1}**H-NMR** (400 MHz; DMSO-d6): *δ*/ppm = 2.20 – 2.10 (m, 2H, P-CH_2), 1.82 (d, *J* = 14.0 Hz, 9H, P-CH_3), 1.58 – 1.35 (m, 2H, P-CH_2-***CH_2*** + ***CH_2***-CH_3), 0.92 (t, *J* = 7.2 Hz, 3H, CH_2-***CH_3***).

^{13}**C{^{1}H}-NMR** (101 MHz; DMSO-d6): *δ*/ppm = 119.8 (q, *J* = 321.6 Hz, CF_3), 23.40 (d, *J* = 16.4 Hz, ***CH_2***-CH_3), 22.62 (d, *J* = 4.3 Hz, P-CH_2-***CH_2***), 22.12(d, *J* = 52.2 Hz, P-CH_2), 13.23 (s, CH_2-***CH_3***), 7.12 (d, *J* = 54.1 Hz, P-CH_3).

^{19}**F{^{1}H}-NMR** (377 MHz; DMSO-d6): *δ*/ppm = -78.89 (s).

^{31}**P{^{1}H}-NMR** (162 MHz; DMSO-d6): *δ*/ppm = 27.85 (s).

1.2.2 Trimethylpentylphosphonium bis(trifluoromethanesulfonyl)imide [P1115][TFSI]/[5a]

^{1}**H-NMR** (400 MHz; DMSO-d6): *δ*/ppm = 2.20 – 2.06 (m, 2H, P-CH_2), 1.81 (d, *J* = 14.8 Hz, 9H, P-CH_3), 1.56 – 1.44 (m, 2H, P-CH_2-***CH_2***), 1.42 – 1.27 (m, 4H, P-CH_2-***CH_2***+ ***CH_2***-CH_3), 0.90 (t, *J* = 6.9 Hz, 3H, CH_2-***CH_3***).

^{13}**C{^{1}H}-NMR** (101 MHz; DMSO-d6): *δ*/ppm = 120.0 (q, *J* = 321.8 Hz, CF_3), 32.10 (d, *J* = 16.1 Hz, P-$(CH_2)_2$-***CH_2***), 22.19 (d, *J* = 52.3 Hz, P-CH_2), 21.39 (s, ***CH_2***-CH_3) 20.24 (d, *J* = 4.3 Hz, P-CH_2-***CH_2***), 13.64 (s, CH_2-***CH_3***), 7.13 (d, *J* = 54.1 Hz, P-CH_3).

^{19}**F{^{1}H}-NMR** (377 MHz; DMSO-d6): *δ*/ppm = -78.86 (s).

^{31}**P{^{1}H}-NMR** (162 MHz; DMSO-d6): *δ*/ppm = 28.84 (s).

1.2.3 (2-methoxyethyl)trimethylphosphonium bis(trifluoromethanesulfonyl)imide [P1112O1][TFSI]/[2O1a]

^{1}**H-NMR** (400 MHz; DMSO-d6): *δ*/ppm = 3.67 (dt, *J* = 19.5 Hz, *J* = 6.1 Hz, 2H, P-CH_2-***CH_2***), 3.29 (s, 3H, O-CH_3), 2.62 (dt, *J* = 13.8 Hz, *J* = 6.1 Hz, 2H, P-***CH_2***-CH_2) 1.83 (t, *J* = 15.0 Hz, 9H, P-CH_3).

^{13}**C{^{1}H}-NMR** (101 MHz; DMSO-d6): *δ*/ppm = 119.5 (q, *J* = 321.8 Hz, CF_3), 64.81 (d, *J* = 6.2 Hz, P-CH_2-***CH_2***), 58.05 (s, O-CH_3), 23.37 (d, *J* = 53.7 Hz, P-CH_2), 8.02 (d, *J* = 54.1 Hz, P-CH_3).

^{19}**F{^{1}H}-NMR** (377 MHz; DMSO-d6): *δ*/ppm = -78.91 (s).

^{31}**P{^{1}H}-NMR** (162 MHz; DMSO-d6): *δ*/ppm = 27.64 (s).

1.2.4 (2-ethoxyethyl)trimethylphosphonium bis(trifluoromethanesulfonyl)imide [P1112O2][TFSI]/[2O2a]

^{1}**H-NMR** (400 MHz; $CDCl_3$): *δ*/ppm = 3.78 (dt, *J* = 21.0 Hz, *J* = 7.0 Hz, 2H, P-CH_2-***CH_2***), 3.52 (q, *J* = 7.0, 2H, ***CH_2***-CH_3), 2.51 (dt, *J* = 12.8 Hz, *J* = 5.9 Hz, 2H, P-***CH_2***-CH_2) 1.89 (t, *J* = 14.3 Hz, 9H, P-CH_3), 1.19 (t, *J* = 7.0 Hz, 3H, O-CH_3).

^{13}**C{^{1}H}-NMR** (101 MHz; $CDCl_3$): *δ*/ppm = 119.9 (q, *J* = 321.0 Hz, CF_3), 67.15 (s, ***CH_2***-CH_3), (d, *J* = 8.26 Hz, P-CH_2-***CH_2***), 24.75 (d, *J* = 55.0 Hz, P-CH_2), 14.99 (s, CH_2-***CH_3***) 9.25 (d, *J* = 55.1 Hz, P-CH_3).

^{19}F{^{1}H}-NMR (377 MHz; $CDCl_3$): δ/ppm = -78.84 (s).

^{31}P{^{1}H}-NMR (162 MHz; $CDCl_3$): δ/ppm = 26.78 (s).

1.3 Synthesis of the phosphonium bis(fluorosulfonyl)imde ionic liquids

The bis(fluorosulfonyl)imide ionic liquids were prepared similar to the bis(trifluoromethane-sulfonyl)imide ionic liquids but with back-extraction of each of the aqueous washings with a slight amount of dichloromethane for two times during each washing step. After drying for two days in a vacuum with stirring, the samples were obtained as slightly yellowish liquids.

1.3.1 Butyltrimethylphosphonium bis(fluorosulfonyl)imide [P1114][FSI]/[4b]

^{1}H-NMR (400 MHz; DMSO-d6): δ/ppm = 2.20 – 2.08 (m, 2H, P-CH_2), 1.80 (d, *J* = 14.8 Hz, 9H, P-CH_3), 1.56 – 1.33 (m, 2H, P-CH_2-***CH_2*** + ***CH_2***-CH_3), 0.91 (t, *J* = 7.2 Hz, 3H, CH_2-***CH_3***).

^{13}C{^{1}H}-NMR (101 MHz; DMSO-d6): δ/ppm = 23.20 (d, *J* = 16.5 Hz, ***CH_2***-CH_3), 22.58 (d, *J* = 4.3 Hz, P-CH_2-***CH_2***), 21.98 (d, *J* = 52.3 Hz, P-CH_2), 13.22 (s, CH_2-***CH_3***), 7.12 (d, *J* = 53.9 Hz, P-CH_3).

^{19}F{^{1}H}-NMR (377 MHz; DMSO-d6): δ/ppm = 53.24 (s).

^{31}P{^{1}H}-NMR (162 MHz; DMSO-d6): δ/ppm = 27.87 (s).

1.3.2 Trimethylpentylphosphonium bis(fluorosulfonyl)imide [P1115][FSI]/[5b]

^{1}H-NMR (400 MHz; DMSO-d6): δ/ppm = 2.19 – 2.07 (m, 2H, P-CH_2), 1.80 (d, *J* = 14.8 Hz, 9H, P-CH_3), 1.55 – 1.43 (m, 2H, P-CH_2-***CH_2***), 1.41 – 1.26 (m, 4H, P-CH_2-***CH_2*** + ***CH_2***-CH_3), 0.89 (t, *J* = 6.9 Hz, 3H, CH_2-***CH_3***).

^{13}C{^{1}H}-NMR (101 MHz; DMSO-d6): δ/ppm = 32.10 (d, *J* = 16.1 Hz, P-$(CH_2)_2$-***CH_2***), 22.18 (d, *J* = 52.3 Hz, P-CH_2), 21.40 (s, ***CH_2***-CH_3) 20.22 (d, *J* = 4.3 Hz, P-CH_2-***CH_2***), 13.61 (s, CH_2-***CH_3***), 7.12 (d, *J* = 54.1 Hz, P-CH_3).

^{19}F{^{1}H}-NMR (377 MHz; DMSO-d6): δ/ppm = 53.22 (s).

^{31}P{^{1}H}-NMR (162 MHz; DMSO-d6): δ/ppm = 28.82 (s).

1.3.3 (2-methoxyethyl)trimethylphosphonium bis(fluorosulfonyl)imide [P1112O1][FSI]/[2O1b]

^{1}H-NMR (400 MHz; DMSO-d6): δ/ppm = 3.67 (dt, *J* = 19.8 Hz, *J* = 6.2 Hz, 2H, P-CH_2-***CH_2***), 3.29 (s, 3H, O-CH_3), 2.54 – 2.45 (m, 2H, P-***CH_2***-CH_2) 1.82 (t, *J* = 15.0 Hz, 9H, P-CH_3).

^{13}C{^{1}H}-NMR (101 MHz; DMSO-d6): δ/ppm = 64.83 (d, *J* = 6.2 Hz, P-CH_2-***CH_2***), 58.09 (s, O-CH_3), 23.41 (d, *J* = 53.8 Hz, P-CH_2), 8.06 (d, *J* = 54.2 Hz, P-CH_3).

^{19}F{^{1}H}-NMR (377 MHz; DMSO-d6): δ/ppm = 53.25 (s).

^{31}P{^{1}H}-NMR (162 MHz; DMSO-d6): δ/ppm = 27.58 (s).

1.3.4 (2-ethoxyethyl)trimethylphosphonium bis(fluorosulfonyl)imide [P1112O2][FSI]/[2O2b]

^{1}H-NMR (400 MHz; DMSO-d6): δ/ppm = 3.71 (dt, *J* = 19.9 Hz, *J* = 6.1 Hz, 2H, P-CH_2-***CH_2***), 3.48 (q, *J* = 7.0, 2H, ***CH_2***-CH_3), 2.55 – 2.45 (m, 2H, P-***CH_2***-CH_2) 1.83 (t, *J* = 15.0 Hz, 9H, P-CH_3), 1.13 (t, *J* = 7.0 Hz, 3H, CH_2-***CH_3***),

^{13}C{^{1}H}-NMR (101 MHz; DMSO-d6): δ/ppm = 65.65 (s, ***CH_2***-CH_3) 62.75 (d, *J* = 6.5 Hz, P-CH_2-***CH_2***), 23.55 (d, *J* = 53.9 Hz, P-CH_2), 14.85 (s, CH_2-***CH_3***), 8.10 (d, *J* = 54.1 Hz, P-CH_3).

^{19}F{^{1}H}-NMR (377 MHz; DMSO-d6): δ/ppm = 53.23 (s).

$^{31}P\{^{1}H\}$**-NMR** (162 MHz; DMSO-d6): δ/ppm = 27.50 (s).

2. Density

Experimental values of the temperature-dependent densities are given in Table S1.

Table S1a. Density values given in g mL^{-1} of the ILs and IL-salt mixtures at the stated temperatures.

Sample	15 °C	20 °C	25 °C	30 °C	35°C	40 °C	45 °C	50 °C	60 °C	70 °C	80 °C	90 °C
[4a]	---[1]	---[1]	---[1]	---[1]	---[1]	1.3720	1.3676	1.3632	1.3546	1.3459	1.3373	1.3288
[4a-025]	---[1]	---[1]	---[1]	---[1]	---[1]	1.3923	1.3878	1.3833	1.3744	1.3656	1.3568	1.3480
[4b]	1.3046	1.3006	1.2966	1.2927	1.2887	1.2848	1.2809	1.2770	1.2693	1.2617	1.2541	1.2467
[4b-025]	1.3204	1.3163	1.3122	1.3082	1.3042	1.3002	1.2963	1.2924	1.2846	1.2768	1.2692	1.2616
[5a]	---[1]	---[1]	---[1]	---[1]	---[1]	1.3409	1.3365	1.3322	1.3236	1.3151	1.3066	1.2982
[5a-025]	---[1]	---[1]	---[1]	---[1]	---[1]	1.3625	1.3581	1.3536	1.3448	1.3359	1.3264	1.3170
[5b]	---[1]	---[1]	1.2661	1.2622	1.2584	1.2546	1.2507	1.2470	1.2394	1.2319	1.2245	1.2172
[5b-025]	---[1]	---[1]	1.2825	1.2785	1.2746	1.2706	1.2667	1.2628	1.2550	1.2471	1.2391	1.2310
[2O1a]	---[1]	---[1]	---[1]	---[1]	---[1]	1.4286	1.4239	1.4192	1.4099	1.4006	1.3915	1.3824
[2O1a-025]	---[1]	---[1]	---[1]	---[1]	1.4513	1.4467	1.4420	1.4372	1.4277	1.4183	1.4090	1.3998
[2O1b]	---[1]	---[1]	---[1]	---[1]	1.3555	1.3513	1.3471	1.3430	1.3348	1.3266	1.3185	1.3106
[2O1b-025]	---[1]	---[1]	---[1]	1.3757	1.3714	1.3672	1.3630	1.3588	1.3504	1.3422	1.3340	1.3259
[2O2a]	1.4117	1.4069	1.4022	1.3976	1.3929	1.3883	1.3836	1.3791	1.3699	1.3609	1.3519	1.3430
[2O2a-025]	1.4310	1.4262	1.4214	1.4166	1.4118	1.4071	1.4024	1.3977	1.3884	1.3792	1.3700	1.3610
[2O2b]	1.3280	1.3238	1.3197	1.3156	1.3115	1.3074	1.3033	1.2992	1.2912	1.2832	1.2753	1.2674
[2O2b-025]	1.3456	1.3413	1.3371	1.3329	1.3288	1.3246	1.3205	1.3164	1.3082	1.3001	1.2921	1.2842

[1] Sample became solid at this temperature, thus a measurement was not possible.

Table S1b. Linear fitting values for density according to equation $d = a \cdot T + b$ (temperature in Kelvin).

Sample	*a*	*standard error of a*	*b*	*standard error of b*	R^2
[4a]	-8.642E-4	1.9E-6	1.6426	6.3E-4	0.99997
[4a-025]	-8.846E-4	1.9E-6	1.6692	6.2E-4	0.99997
[4b]	-7.728E-4	2.6E-6	1.5270	8.2E-4	0.99988
[4b-025]	-7.840E-4	2.6E-6	1.5459	8.4E-4	0.99988
[5a]	-8.527E-4	1.8E-6	1.6078	6.1E-4	0.99997
[5a-025]	-9.077E-4	6.3E-6	1.6470	2.11E-3	0.99971
[5b]	-7.523E-4	2.1E-6	1.4902	6.9E-4	0.99993
[5b-025]	-7.897E-4	1.7E-6	1.5180	5.6E-4	0.99996
[2O1a]	-9.241E-4	2.3E-6	1.7178	7.6E-4	0.99996
[2O1a-025]	-9.389E-4	1.7E-6	1.7406	5.7E-4	0.99998
[2O1b]	-8.172E-4	2.3E-6	1.6072	7.7E-4	0.99994
[2O1b-025]	-8.302E-4	2.6E-6	1.6272	8.4E-4	0.99992
[2O2a]	-9.160E-4	2.7E-6	1.6753	8.7E-4	0.99990
[2O2a-025]	-9.344E-4	2.9E-6	1.6999	9.3E-4	0.99989
[2O2b]	-8.082E-4	2.4E-6	1.5606	7.6E-4	0.99990
[2O2b-025]	-8.189E-4	2.7E-6	1.5812	8.8E-4	0.99988

3. Viscosity

Experimental values of the temperature-dependent viscosity are given in Table S2.

Table S2. Viscosity values η in mPa s of the ILs and IL-salt mixtures at the given temperatures. The measurement error related to calibration compounds (viscosity standards) is < 1 % of the viscosity value.

Sample	25 °C	30 °C	35 °C	40 °C	45 °C	50 °C	55 °C	60 °C	65 °C	70 °C	75 °C	80 °C	85 °C	90 °C	95 °C	100 °C	105 °C
[4a]	112	86.4	67.7	54.2	43.8	36.1	30.0	25.4	21.7	18.7	16.3	14.3	12.6	11.2	10.1	9.06	8.13
[4a-025]	162	122	93.1	72.8	57.9	46.9	38.5	32.1	27.0	23.1	19.9	17.2	15.1	13.4	11.9	10.6	9.47
[4b]	44.1	36.7	31.1	26.5	22.9	19.9	17.4	15.4	13.7	12.2	11.0	9.92	9.00	8.21	7.53	6.91	6.38
[4b-025]	53.8	44.4	37.1	31.5	26.9	23.2	20.3	17.8	15.8	14.0	12.6	11.3	10.3	9.36	8.57	7.86	7.23
[5a]	137	103	79.3	62.3	49.8	40.5	33.3	27.8	23.4	20.0	17.2	14.9	13.1	11.5	10.2	9.14	8.22
[5a-025]	199	146	110	84.8	66.5	53.1	43.0	43.0	29.6	24.9	21.3	18.3	15.9	13.9	12.2	10.9	9.71
[5b]	63.3	52.8	43.3	36.0	30.3	25.8	22.2	19.4	17.0	15.0	13.4	12.0	10.8	9.76	8.87	8.09	7.41
[5b-025]	68.9	55.8	46.0	38.5	32.5	27.8	23.9	20.8	18.3	16.1	14.3	12.8	11.5	10.4	9.48	8.65	7.94
[2O1a]	54.8	44.2	36.2	30.1	25.3	21.5	18.5	16.1	14.1	12.4	11.0	9.87	8.89	8.06	7.36	6.70	6.15
[2O1a-025]	72.1	57.3	46.4	38.2	31.8	26.8	22.8	19.6	17.0	14.9	13.2	11.7	10.5	9.51	8.64	7.86	7.13
[2O1b]	30.7	26.0	22.4	19.3	16.9	14.9	13.2	11.8	10.6	9.58	8.68	7.91	7.23	6.64	6.14	5.68	5.27
[2O1b-025]	36.5	30.7	26.2	22.6	19.6	17.2	15.1	13.5	12.1	10.9	9.83	8.94	8.16	7.50	6.90	6.39	5.95
[2O2a]	53.3	42.8	34.9	29.0	24.3	20.6	17.7	15.4	13.4	11.8	10.5	9.41	8.46	7.65	6.93	6.32	5.78
[2O2a-025]	74.4	58.7	47.1	38.5	31.8	26.7	22.6	19.4	16.8	14.6	12.8	11.4	10.1	9.08	8.17	7.40	6.75
[2O2b]	33.8	28.5	24.4	21.0	18.3	16.0	14.1	12.6	11.3	10.1	9.16	8.32	7.60	6.98	6.43	5.93	5.50
[2O2b-025]	37.3	31.3	26.6	22.8	19.8	17.3	15.2	13.5	12.1	10.9	9.82	8.91	8.12	7.44	6.83	6.27	5.81

4. Specific conductivity

Experimental values of the temperature-dependent specific conductivity are given in Table S3.

Table S3. Specific conductivity values κ in mS cm^{-1} of the ILs and IL-salt mixtures at the given temperatures. The measurement error related to calibration compounds (conductivity standards) is < 2 % of the viscosity value.

Sample	10 °C	15 °C	20 °C	25 °C	30 °C	35 °C	40 °C	45 °C	50 °C	55 °C	60 °C	65 °C	70 °C	75 °C	80 °C	85 °C
[4a]	0.763	1.03	1.38	1.76	2.24	2.78	3.42	4.14	4.92	5.79	6.74	7.78	8.89	10.1	11.4	12.7
[4a-025]	---[1]	0.677	0.936	1.24	1.61	2.04	2.54	3.11	3.77	4.50	5.29	6.17	7.13	8.16	9.25	10.5
[4b]	3.77	4.56	5.47	6.49	7.61	8.82	10.1	11.5	13.0	14.6	16.2	18.0	19.8	21.6	23.6	25.6
[4b-025]	3.15	3.87	4.68	5.57	6.58	7.67	8.86	10.1	11.5	13.0	14.5	16.1	17.8	19.6	21.5	23.5
[5a]	---[1]	0.676	0.913	1.20	1.55	1.95	2.42	2.97	3.59	4.27	5.01	5.82	6.72	7.68	8.70	9.78
[5a-025]	0.334	0.475	0.658	0.889	1.17	1.50	1.89	2.34	2.87	3.46	4.11	4.82	5.58	6.43	7.36	8.32
[5b]	2.22	2.77	3.43	4.16	4.97	5.86	6.85	7.93	9.08	10.3	11.7	13.1	14.5	16.1	17.7	19.4
[5b-025]	1.96	2.45	3.05	3.73	4.49	5.32	6.24	7.26	8.35	9.52	10.8	12.1	13.5	15	16.5	18.1
[2O1a]	1.67	2.15	2.71	3.35	4.08	4.86	5.73	6.69	7.73	8.82	10.0	11.4	12.7	14.1	15.5	17.1
[2O1a-025]	1.21	1.58	2.02	2.53	3.13	3.82	4.56	5.41	6.26	7.24	8.23	9.40	10.6	11.9	13.3	14.6
[2O1b]	4.69	5.61	6.64	7.79	9.02	10.4	11.8	13.3	14.8	16.6	18.2	20.0	22.0	24	26.0	28.2
[2O1b-025]	4.09	4.93	5.87	6.94	8.09	9.33	10.7	12.1	13.6	15.2	16.8	18.6	20.4	22.3	24.2	26.2
[2O2a]	1.43	1.85	2.33	2.89	3.53	4.24	5.01	5.86	6.79	7.78	8.84	9.98	11.2	12.4	13.8	15.1
[2O2a-025]	1.04	1.36	1.75	2.20	2.73	3.33	4.00	4.73	5.51	6.38	7.33	8.33	9.39	10.5	11.7	12.9
[2O2b]	4.21	5.05	6.00	7.07	8.21	9.44	10.8	12.2	13.7	15.2	16.8	18.5	20.3	22.2	24.1	26.0
[2O2b-025]	3.68	4.46	5.29	6.14	7.31	8.51	9.70	10.9	12.1	13.6	15.1	16.7	18.3	20.1	21.9	23.9

[1] Sample became solid at this temperature, thus a measurement was not possible.

5. Molar conductivity

Experimental values of the temperature-dependent molar conductivity as calculated from the specific conductivity and the density of the samples are given in Table S4.

Table S4. Molar conductivity values of the ILs and IL-salt mixtures given in S cm^2 mol^{-1} at the given temperatures.

Sample	10 °C	20 °C	30 °C	40 °C	50 °C	60 °C	70 °C	80 °C
[4a]	0.76	1.38	2.24	3.42	4.92	6.74	8.89	11.35
[4a-025]	--- [1]	0.94	1.61	2.54	3.77	5.29	7.13	9.25
[4b]	--- [1]	0.91	1.55	2.42	3.59	5.01	6.72	8.70
[4b-025]	0.33	0.66	1.17	1.89	2.87	4.11	5.58	7.36
[5a]	3.77	5.47	7.61	10.13	13.03	16.23	19.77	23.58
[5a-025]	3.15	4.67	6.58	8.86	11.51	14.49	17.79	21.49
[5b]	2.22	3.43	4.97	6.85	9.08	11.65	14.53	17.67
[5b-025]	1.95	3.05	4.49	6.24	8.35	10.78	13.52	16.49
[2O1a]	1.67	2.71	4.08	5.73	7.73	10.01	12.65	15.53
[2O1a-025]	1.21	2.02	3.13	4.56	6.26	8.22	10.58	13.32
[2O1b]	1.43	2.33	3.53	5.01	6.79	8.84	11.18	13.76
[2O1b-025]	1.04	1.75	2.73	4.00	5.51	7.33	9.39	11.74
[2O2a]	4.69	6.64	9.02	11.78	14.84	18.22	21.98	26.01
[2O2a-025]	4.09	5.87	8.09	10.66	13.6	16.82	20.36	24.19
[2O2b]	4.21	6.00	8.21	10.77	13.66	16.82	20.30	24.06
[2O2b-025]	3.68	5.29	7.31	9.7	12.18	15.11	18.34	21.88

[1] Sample became solid at this temperature, thus a measurement was not possible.

6. Walden relation

Fitting paramterers for the Walden euqation (SX) are given in Table S5.

$$\log(\Lambda_{\mathrm{m}}/\mathrm{S\ cm^2\ mol^{-1}}) = \log(C) + t\log(\eta^{-1}/\ (0.1\ \mathrm{Pa\ s})^{-1}) \qquad \text{(S-Eq-1)}$$

Table S5. Fitting parameters for the Walden relation following equation S-Eq-1.

Sample	$log(C)$	t	R^2
[4a]	-0.2320 ± 0.0011	0.9167 ± 0.0019	0.99998
[4a-025]	-0.2599 ± 0.0006	0.9162 ± 0.0012	0.99999
[4b]	-0.1201 ± 0.0001	0.8885 ± 0.0014	0.99998
[4b-025]	-0.1269 ± 0.0001	0.8876 ± 0.0016	0.99998
[5a]	-0.2990 ± 0.0009	0.9122 ± 0.0016	0.99999
[5a-025]	-0.3018 ± 0.0022	0.9024 ± 0.0050	0.99988
[5b]	-0.1397 ± 0.0023	0.8818 ± 0.0039	0.99986
[5b-025]	-0.1813 ± 0.0007	0.9068 ± 0.0012	0.99999
[2O1a]	-0.2587 ± 0.0017	0.9187 ± 0.0022	0.99998
[2O1a-025]	-0.2796 ± 0.0040	0.9238 ± 0.0063	0.99977
[2O1b]	-0.2126 ± 0.0014	0.9130 ± 0.0016	0.99998
[2O1b-025]	-0.2099 ± 0.0006	0.9121 ± 0.0008	>0.99999
[2O2a]	-0.3043 ± 0.0009	0.9190 ± 0.0013	0.99999
[2O2a-025]	-0.3030 ± 0.0009	0.9080 ± 0.0016	0.99998
[2O2b]	-0.1756 ± 0.0009	0.8974 ± 0.0011	0.99999
[2O2b-025]	-0.2113 ± 0.0050	0.9002 ± 0.0067	0.99961

7. Polarization ramping

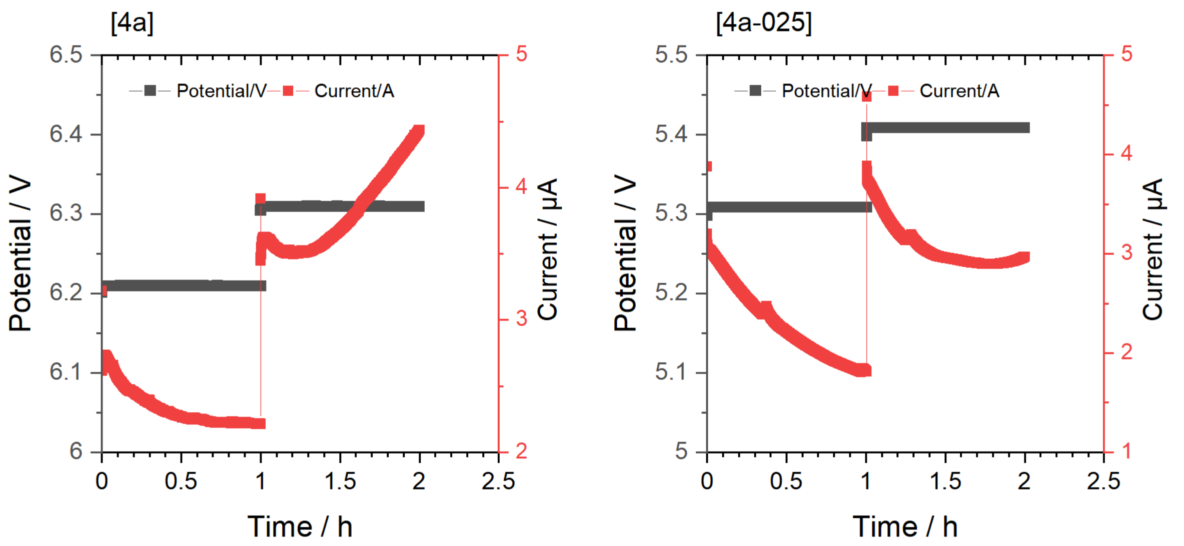

(a) (**b**)

Figure S1. Polarization ramping to obtain the potential limit $U_{ramping}$ in V for [4a] (a) and [4a-025].

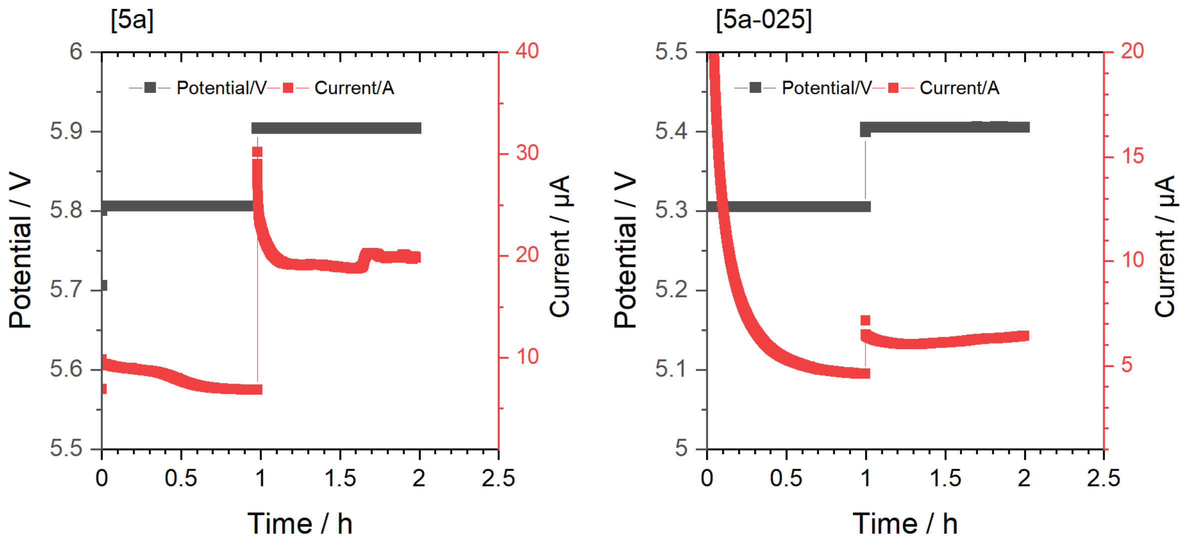

(a) (**b**)

Figure S2. Polarization ramping to obtain the potential limit $U_{ramping}$ in V for [5a] (a) and [5a-025].

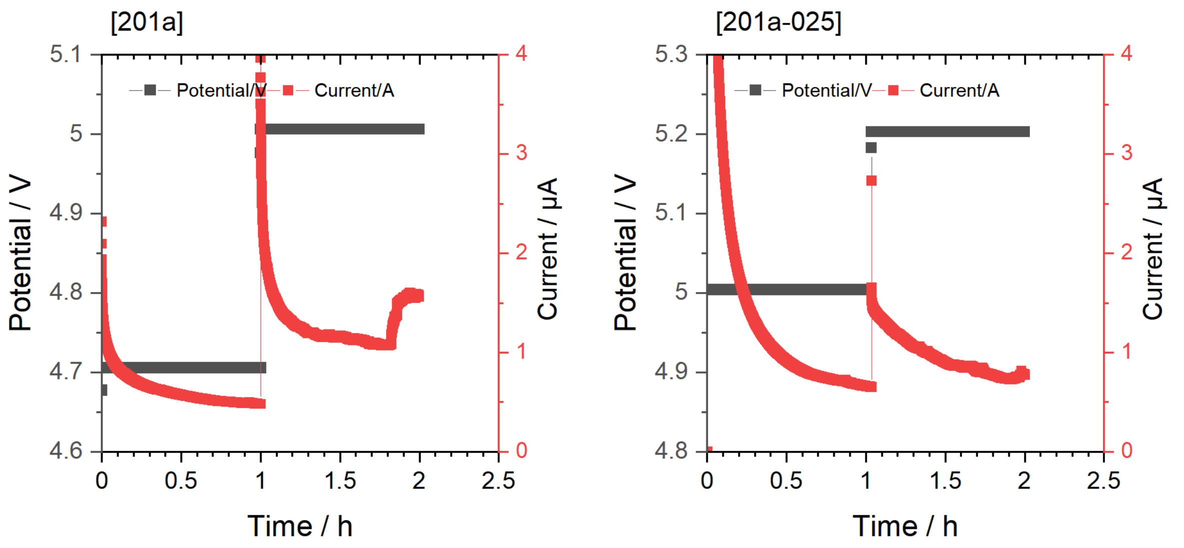

(a) (**b**)

Figure S3. Polarization ramping to obtain the potential limit U_{ramping} in V for [201a] (a) and [201a-025].

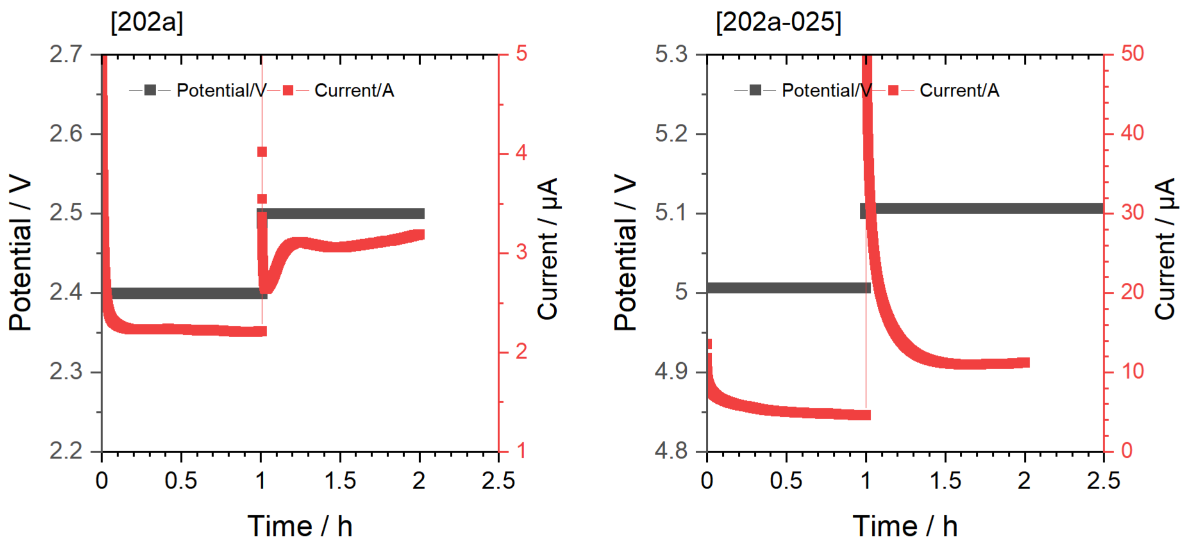

(a) (**b**)

Figure S4. Polarization ramping to obtain the potential limit U_{ramping} in V for [202a] (a) and [202a-025].

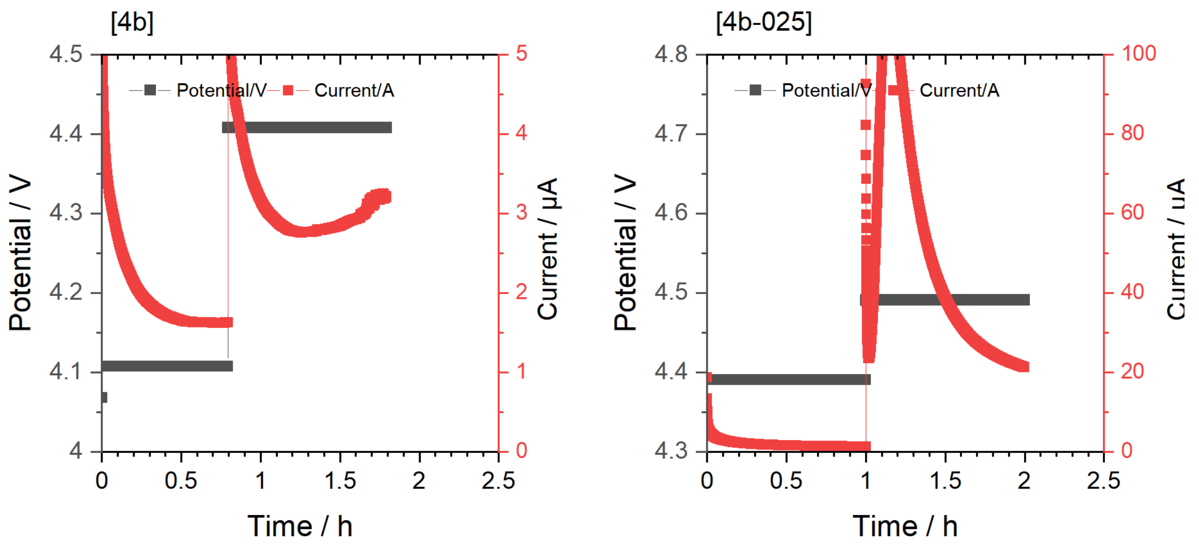

(a) (b)

Figure S5. Polarization ramping to obtain the potential limit $U_{ramping}$ in V for [4b] (a) and [4b-025].

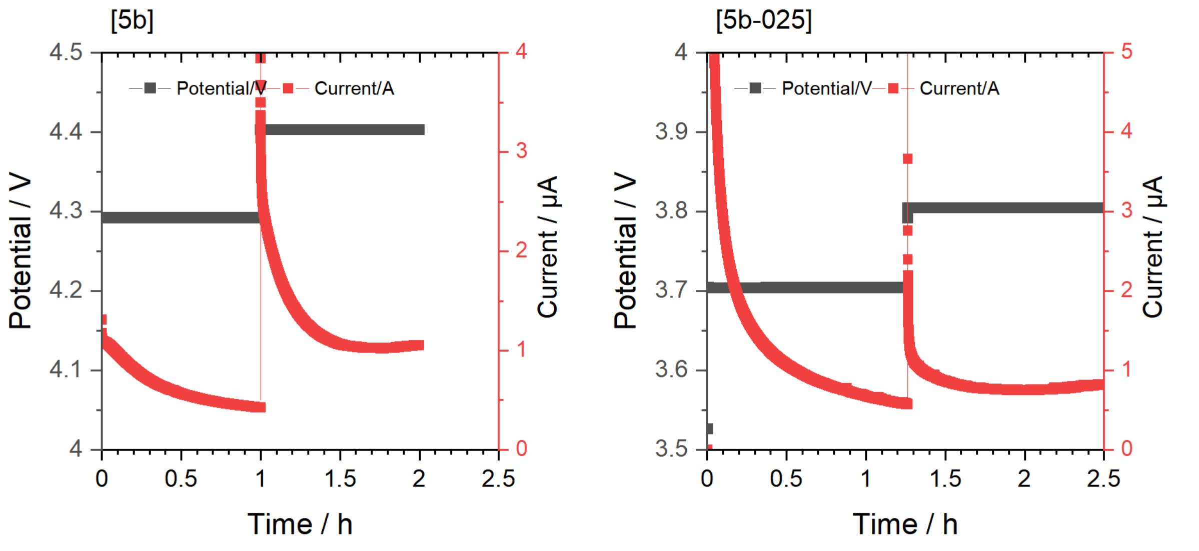

(a) (b)

Figure S6. Polarization ramping to obtain the potential limit $U_{ramping}$ in V for [5b] (a) and [5b-025].

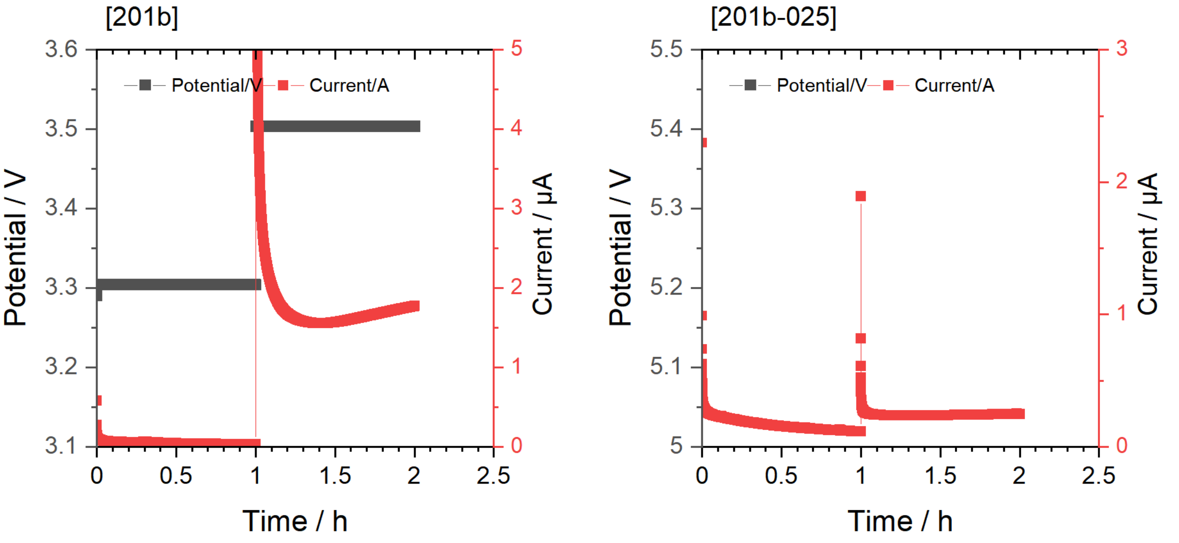

(a) (**b**)

Figure S7. Polarization ramping to obtain the potential limit $U_{ramping}$ in V for [201b] (a) and [201b-025].

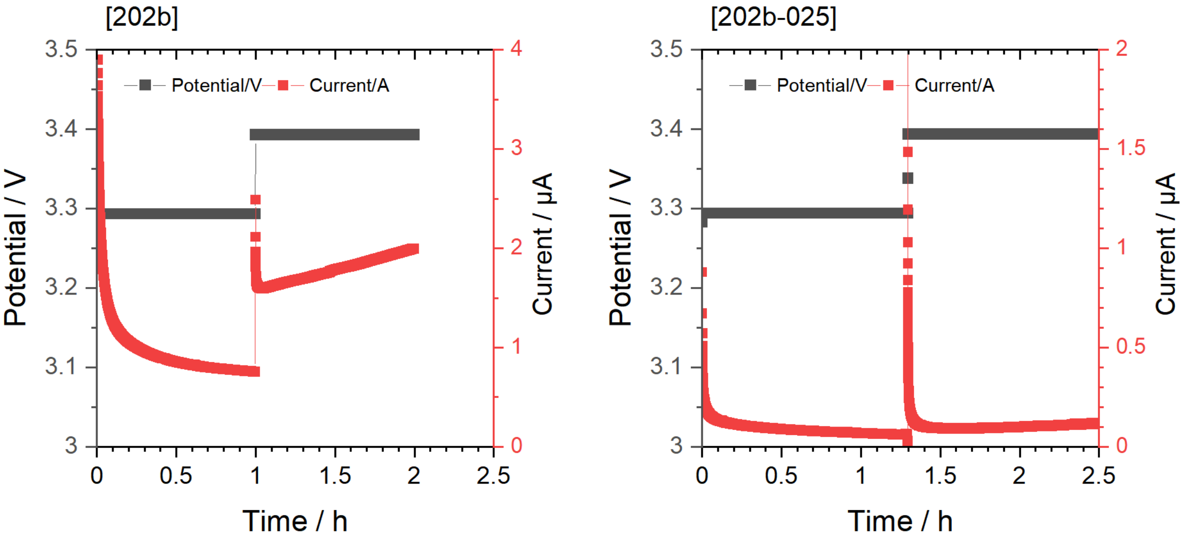

(a) (**b**)

Figure S8. Polarization ramping to obtain the potential limit $U_{ramping}$ in V for [202b] (a) and [202b-025].

8. Microscopy

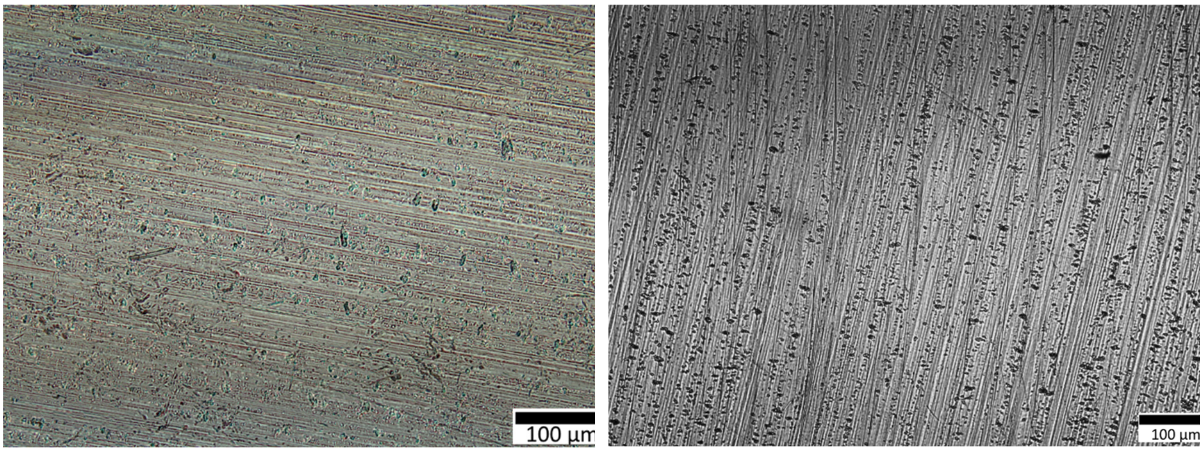

(a) (**b**)

Figure S9. Microscopy images of the samples [4a] (a) and [4a-025] (b) after 30 h polarization at $U_{max,\ pol}$.

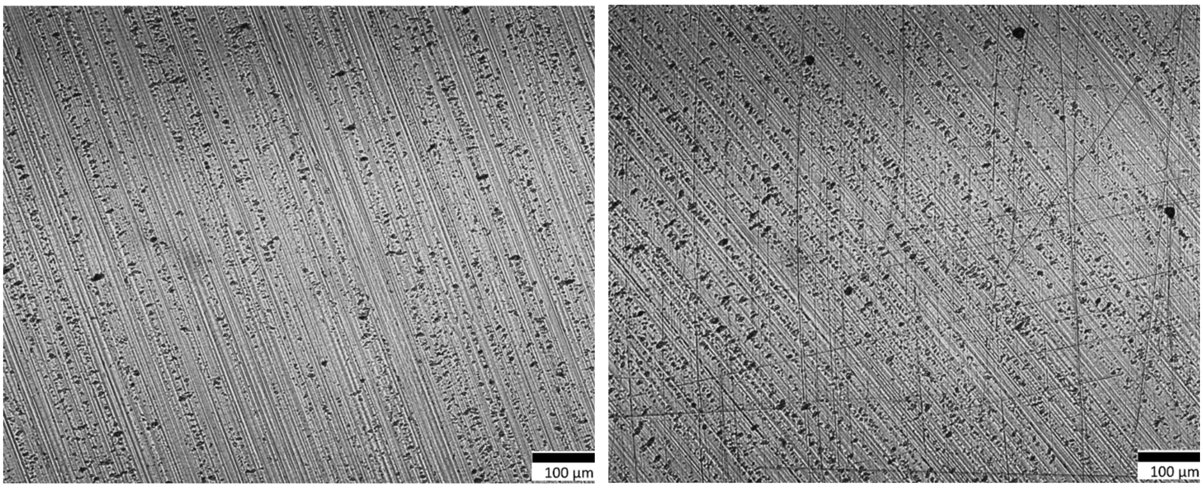

(a) (**b**)

Figure S10. Microscopy images of the samples [5a] (a) and [5a-025] (b) after 30 h polarization at $U_{max,\ pol}$.

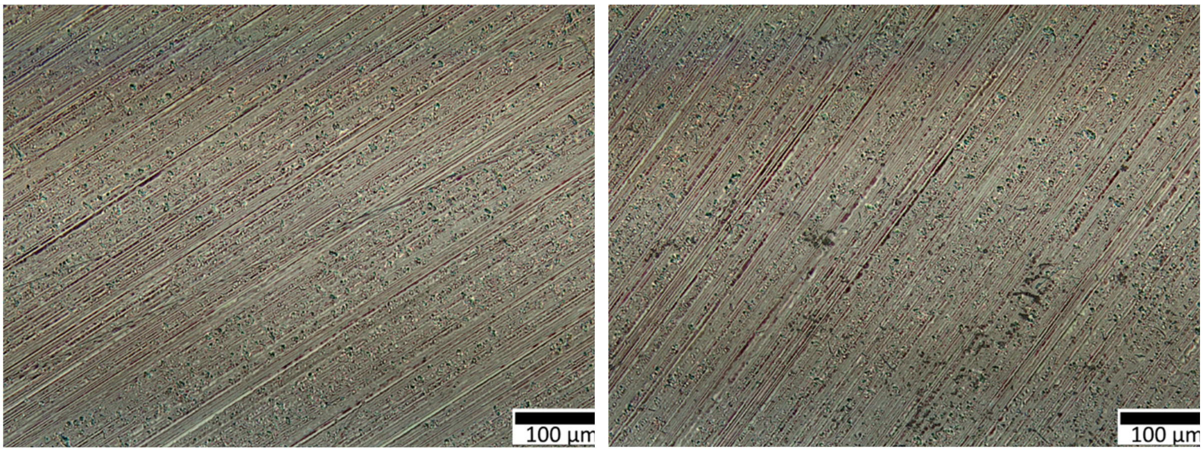

(a) (**b**)

Figure S11. Microscopy images of the samples [201a] (a) and [201a-025] (b) after 30 h polarization at $U_{max,\ pol}$.

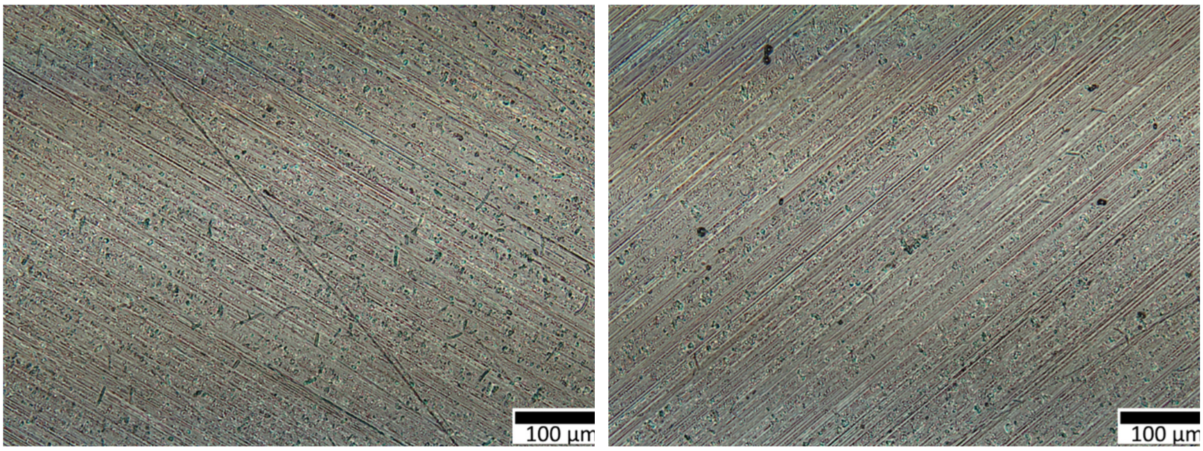

(a) (**b**)

Figure S12. Microscopy images of the samples [202a] (a) and [202a-025] (b) after 30 h polarization at $U_{max, pol}$.

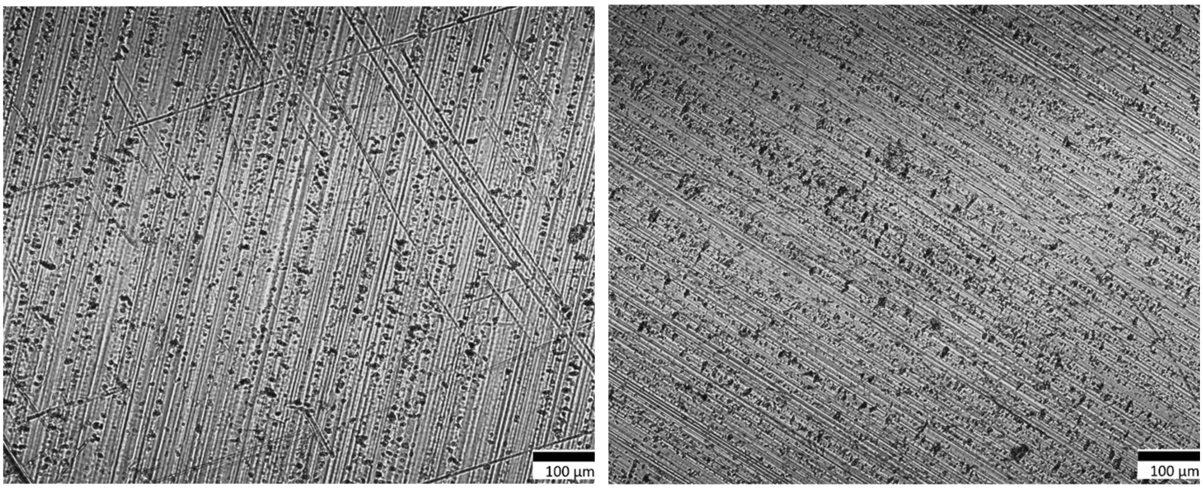

(a) (**b**)

Figure S13. Microscopy images of the samples [4b] (a) and [4b-025] (b) after 30 h polarization at $U_{max, pol}$.

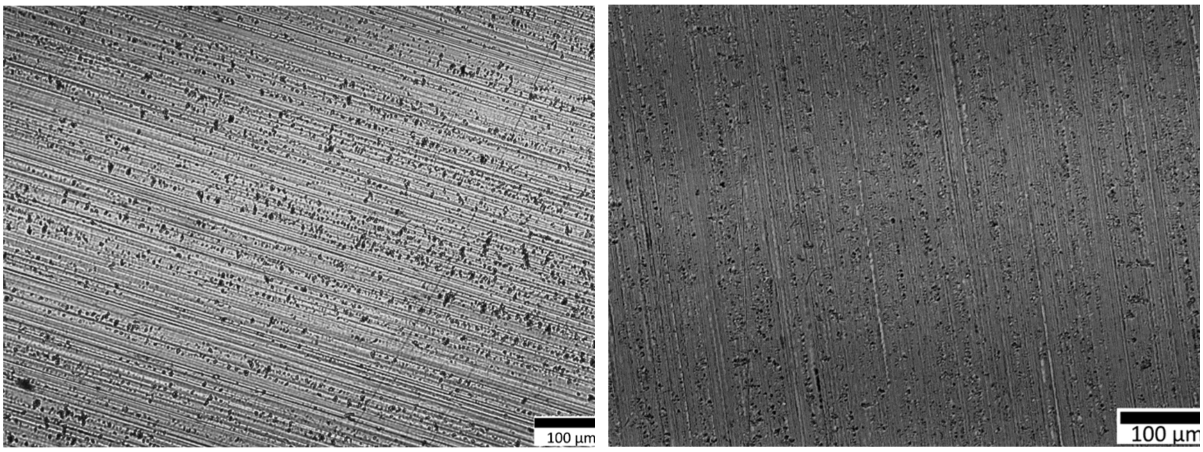

(a) (**b**)

Figure S14. Microscopy images of the samples [5b] (a) and [5b-025] (b) after 30 h polarization at $U_{max, pol}$.

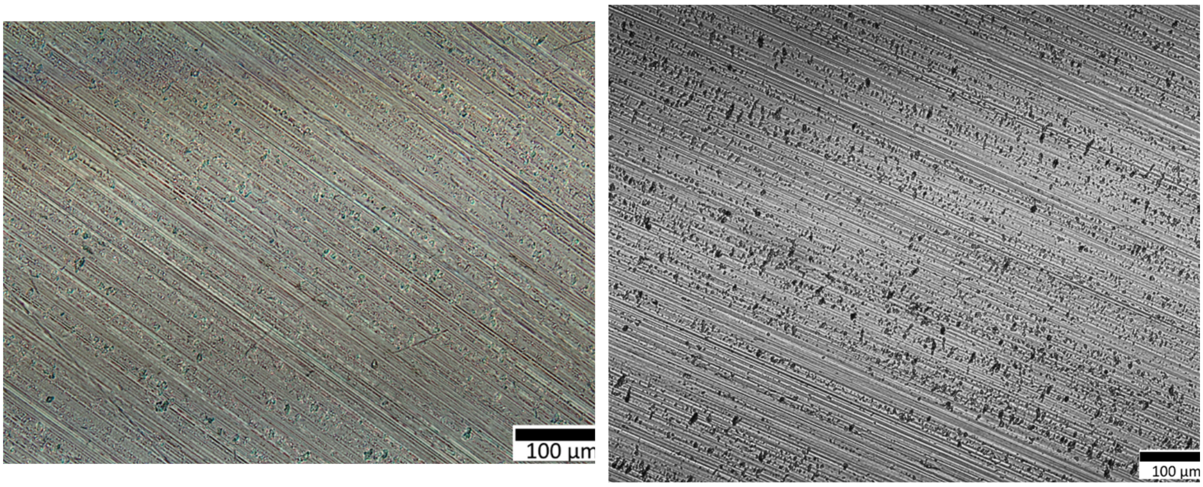

(a) (**b**)

Figure S15. Microscopy images of the samples [201b] (a) and [201b-025] (b) after 30 h polarization at $U_{max, pol}$.

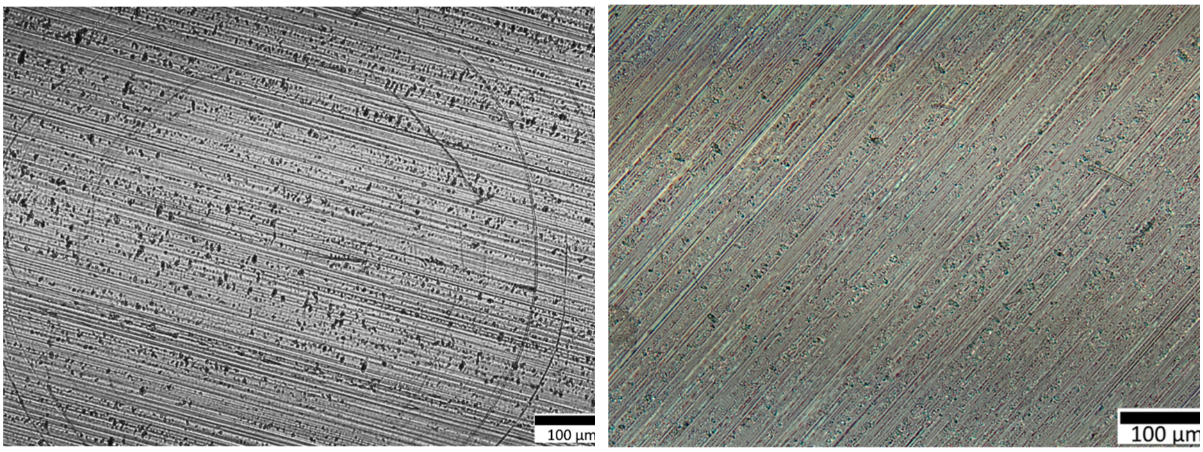

(a) (**b**)

Figure S16. Microscopy images of the samples [202b] (a) and [202b-025] (b) after 30 h polarization at $U_{max, pol}$.

9. Cyclic voltammetry

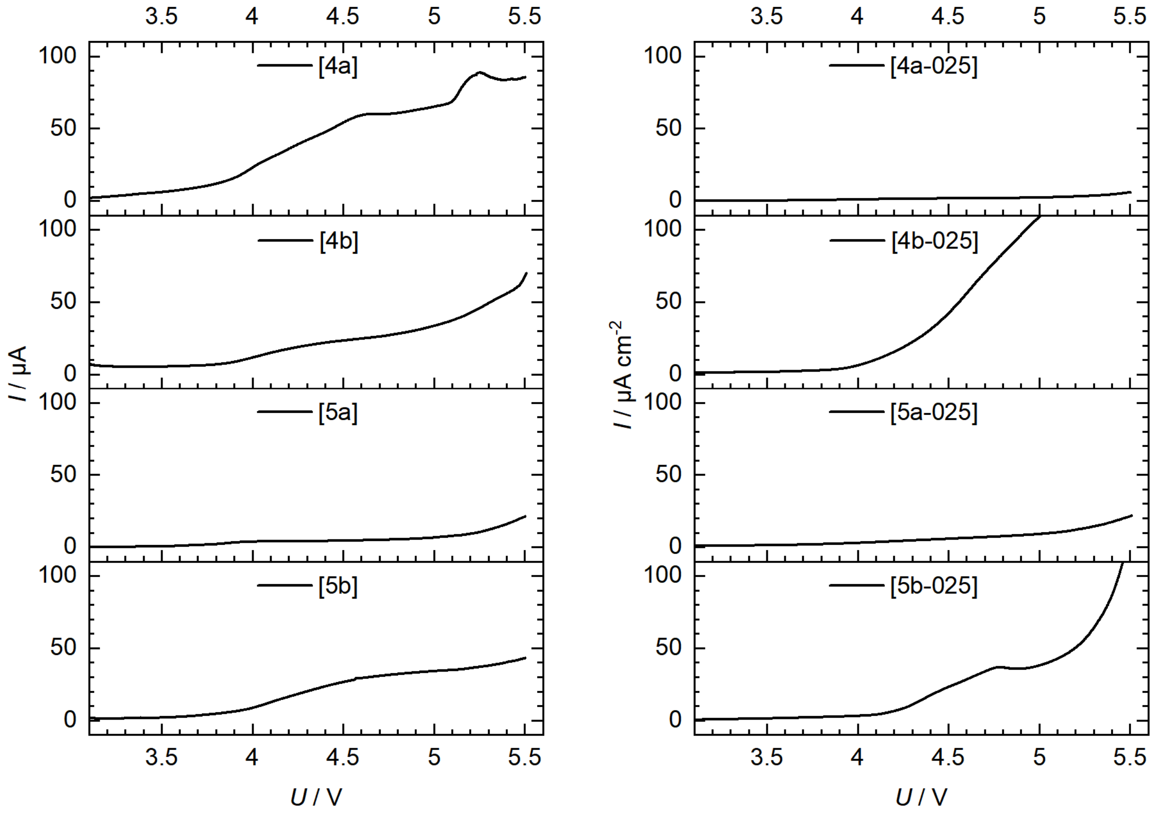

a) (b)

Figure S17. Linear voltammetry scan of Al vs. Li cells from 3.0 to 5.5 V vs. Li/Li$^+$ of (**a**) *n*-alkyl phosphonium ionic liquids and (**b**) *n*-alkyl phosphonium ionic liquid salt mixtures.

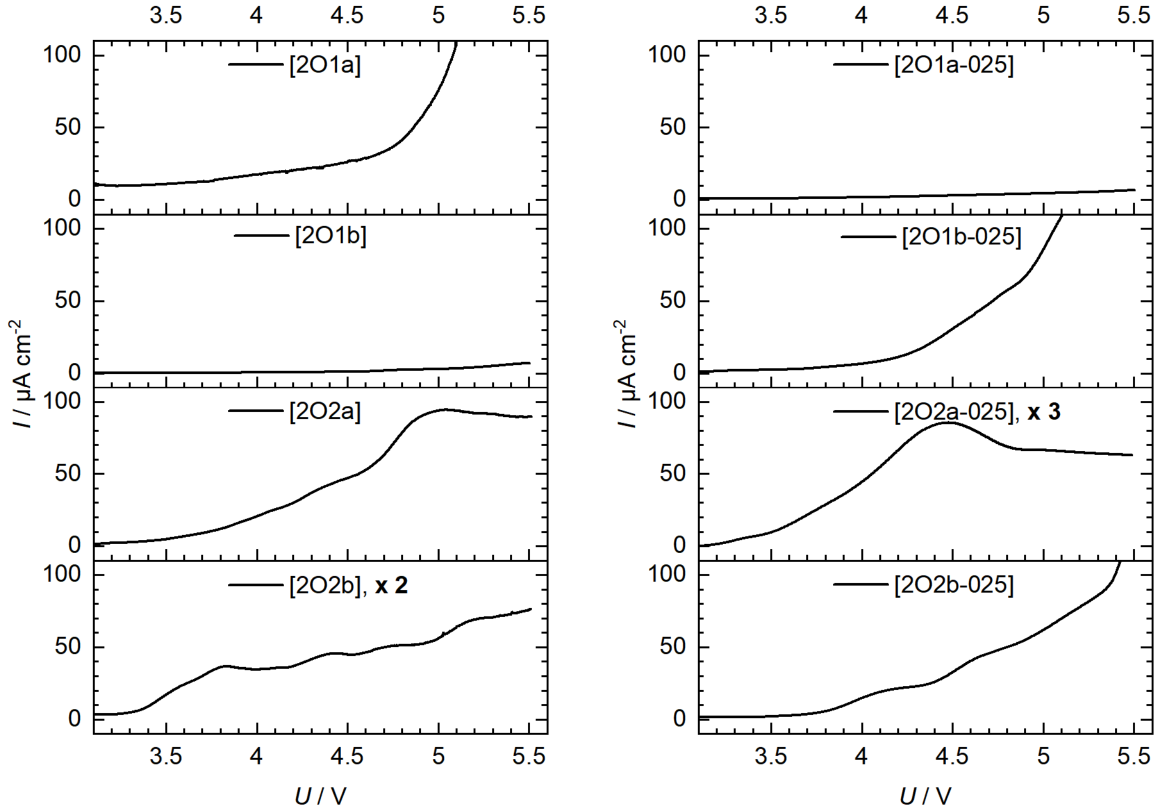

a) (b)

Figure S18. Linear voltammetry scan of Al vs. Li cells from 3.0 to 5.5 V vs. Li/Li$^+$ of (**a**) ether containing phosphonium ionic liquids and (**b**) ether containing phosphonium ionic liquid salt mixtures. Please note that the current density values of [202b] and [202a-025] were reduced by factor 2 or 3 in order to scale all graphs identical.

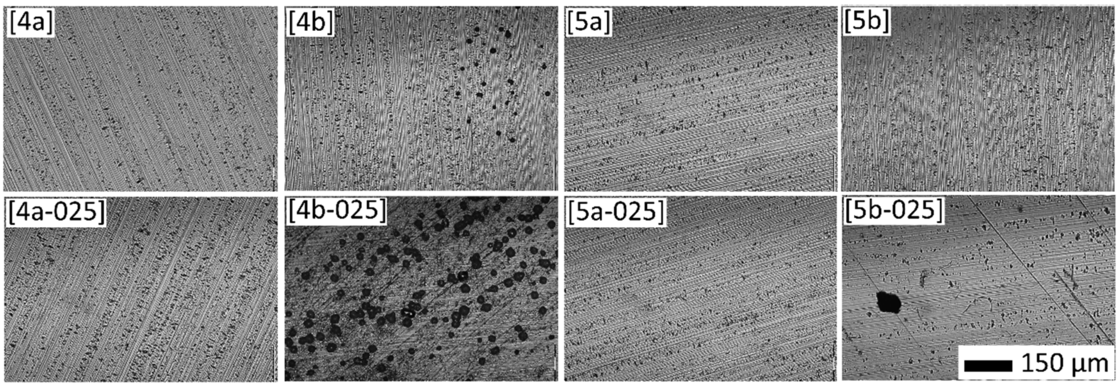

Figure S19. Microscopy images of the aluminum sheets including alcyl phosphonium IL and IL/salt electrolytes after the CV experiment.

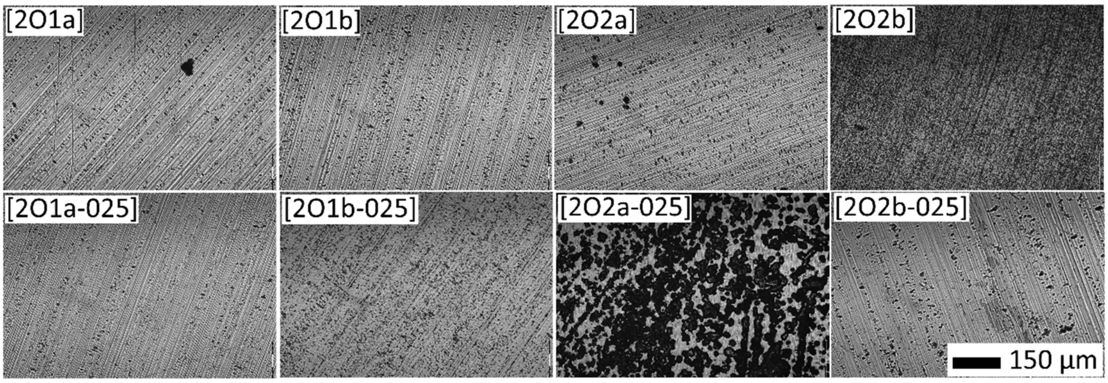

Figure S20. Microscopy images of the aluminum sheets including ether containing phosphonium IL and IL/salt electrolytes after the CV experiment.

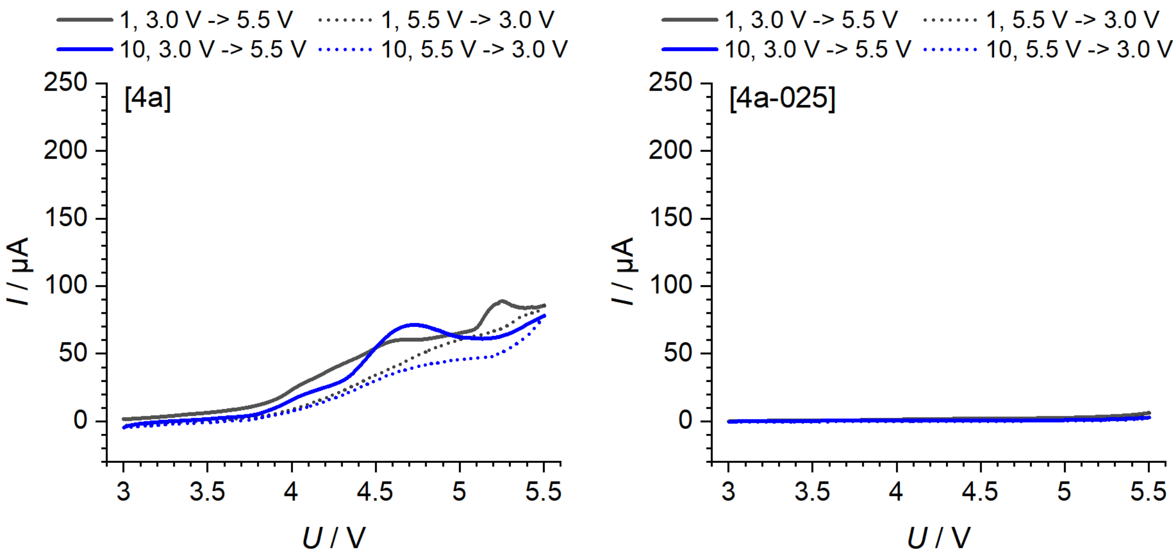

(a) (**b**)

Figure S21. Cyclic voltammetry (CV) measurement of Al//Li cells between 3.0 - 5.5 V vs. Li/Li$^+$ of cycle 1 and cycle 10 of electrolyte [4a] (a) and electrolyte [4a_025] (b).

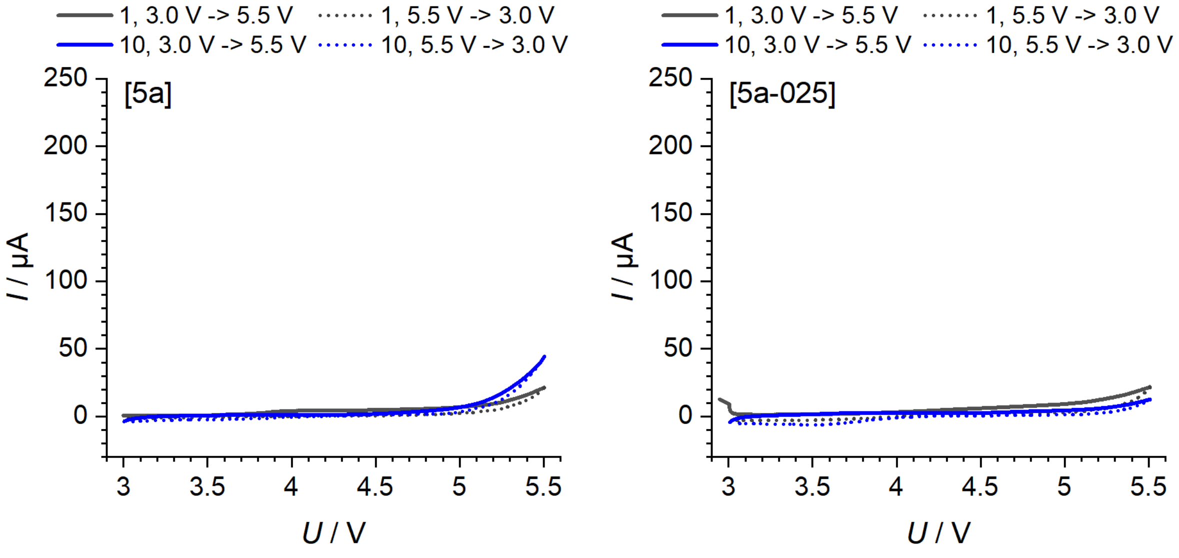

(a) (b)

Figure S22. Cyclic voltammetry (CV) measurement of Al//Li cells between 3.0 - 5.5 V vs. Li/Li$^+$ of cycle 1 and cycle 10 of electrolyte [5a] (a) and electrolyte [5a_025] (b).

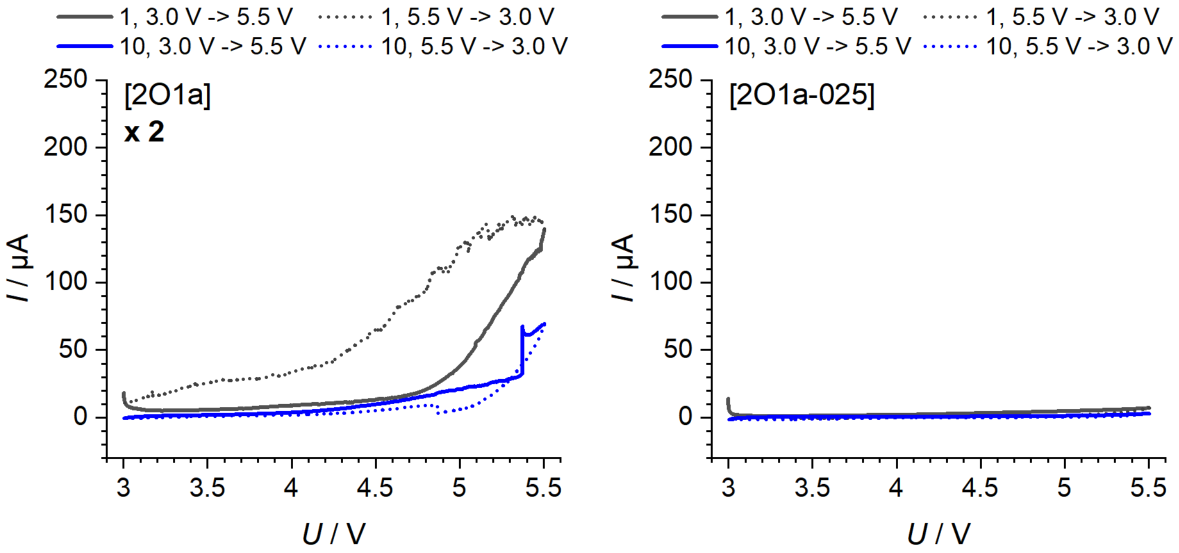

(a) (b)

Figure S23. Cyclic voltammetry (CV) measurement of Al//Li cells between 3.0 - 5.5 V vs. Li/Li$^+$ of cycle 1 and cycle 10 of electrolyte [2O1a] (a) and electrolyte [2O1a_025] (b).

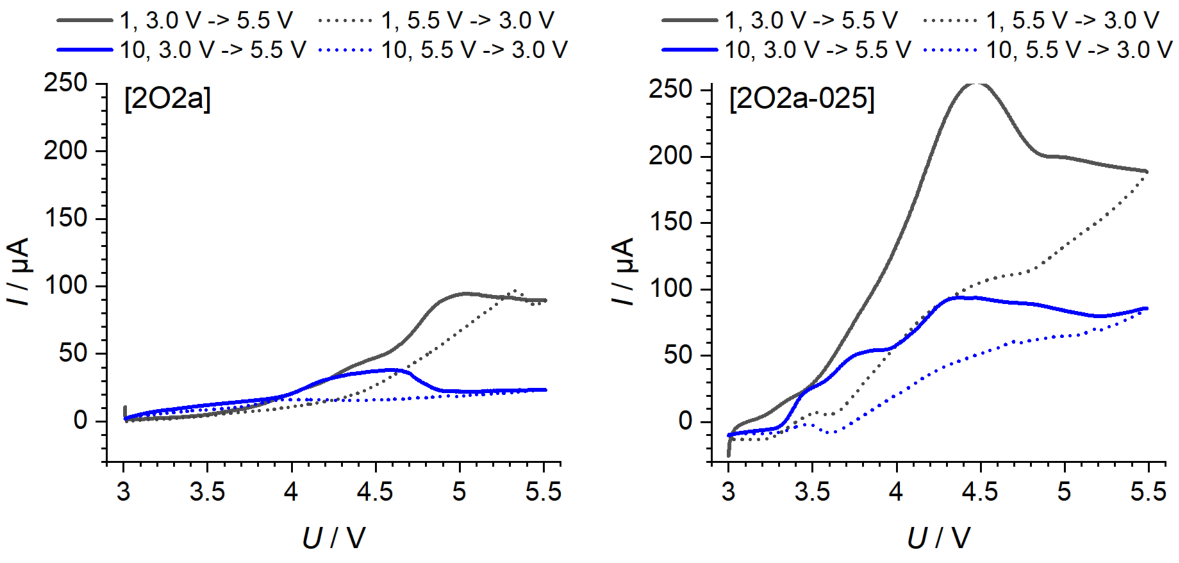

(a) (**b**)

Figure S24. Cyclic voltammetry (CV) measurement of Al//Li cells between 3.0 - 5.5 V vs. Li/Li^{+} of cycle 1 and cycle 10 of electrolyte [2O2a] (a) and electrolyte [2O2a_025] (b).

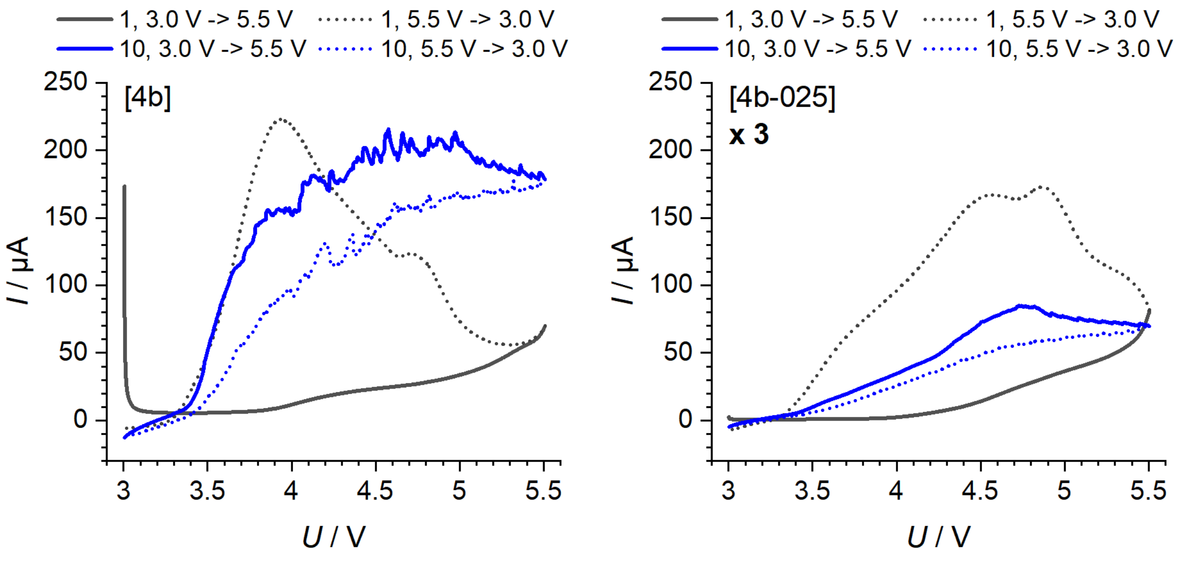

(a) (**b**)

Figure S25. Cyclic voltammetry (CV) measurement of Al//Li cells between 3.0 - 5.5 V vs. Li/Li^{+} of cycle 1 and cycle 10 of electrolyte [4b] (a) and electrolyte [4b_025] (b).

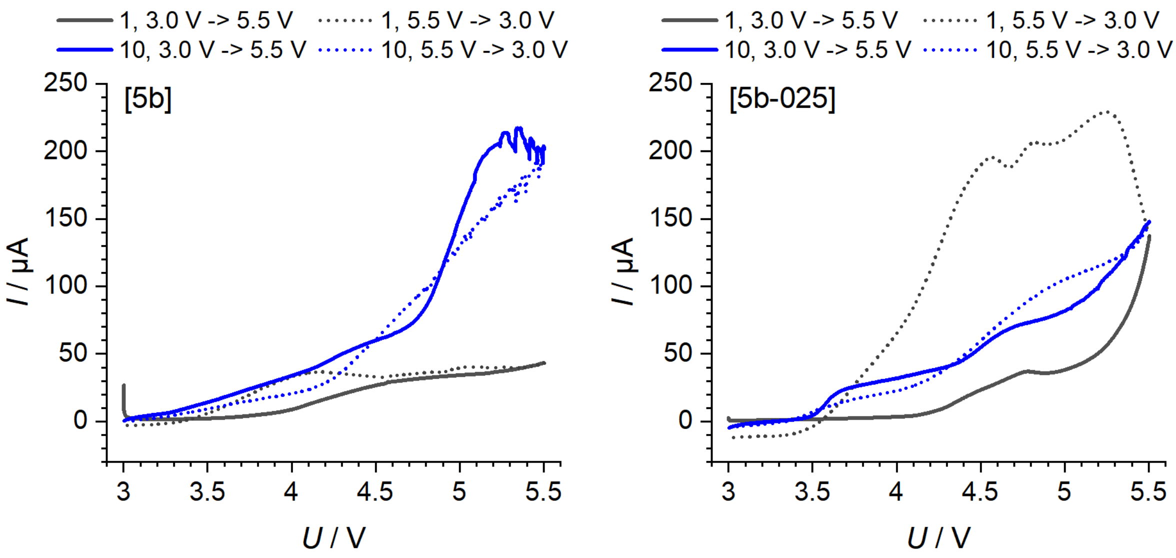

(a) (**b**)

Figure S26. Cyclic voltammetry (CV) measurement of Al//Li cells between 3.0 - 5.5 V vs. Li/Li^+ of cycle 1 and cycle 10 of electrolyte [5b] (a) and electrolyte [5b_025] (b).

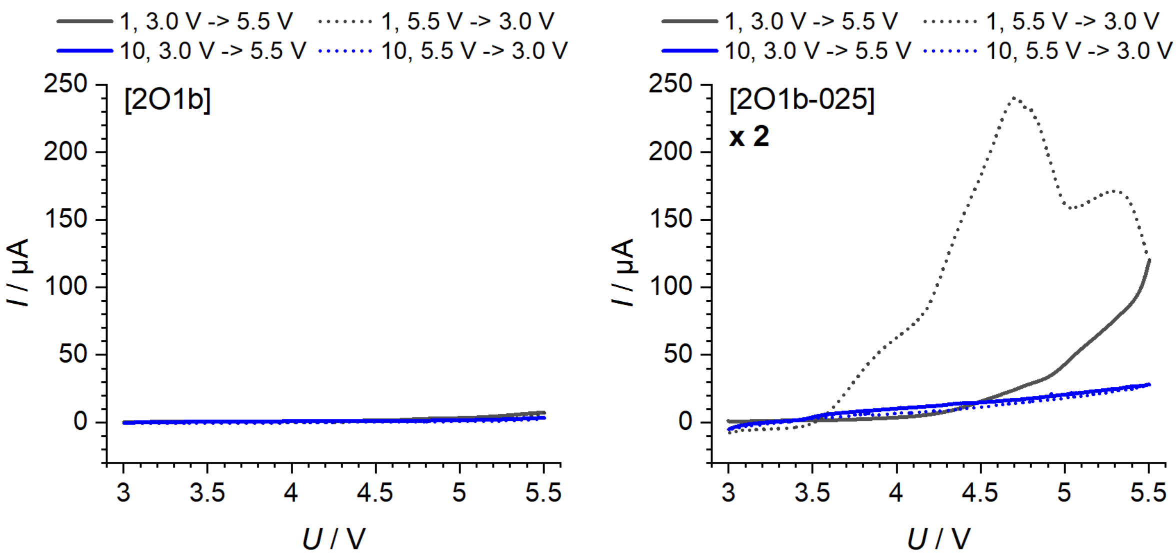

(a) (**b**)

Figure S27. Cyclic voltammetry (CV) measurement of Al//Li cells between 3.0 - 5.5 V vs. Li/Li^+ of cycle 1 and cycle 10 of electrolyte [2O1b] (a) and electrolyte [2O1b_025] (b).

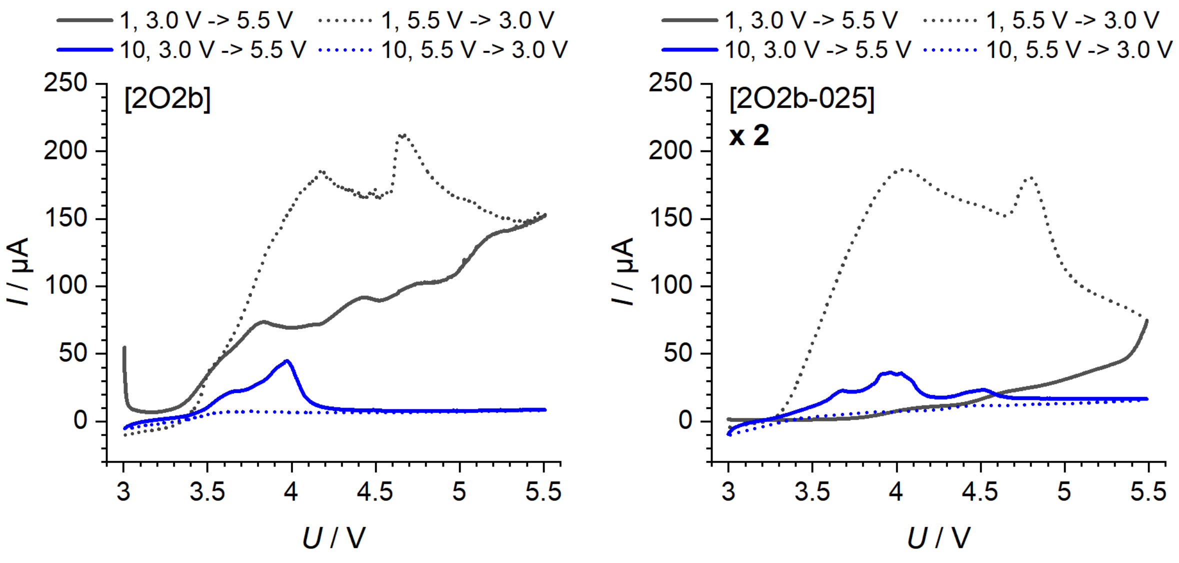

(a) (**b**)

Figure S28. Cyclic voltammetry (CV) measurement of Al//Li cells between 3.0 - 5.5 V vs. Li/Li+ of cycle 1 and cycle 10 of electrolyte [2O2b] (a) and electrolyte [2O2b_025] (b).

10. Battery tests

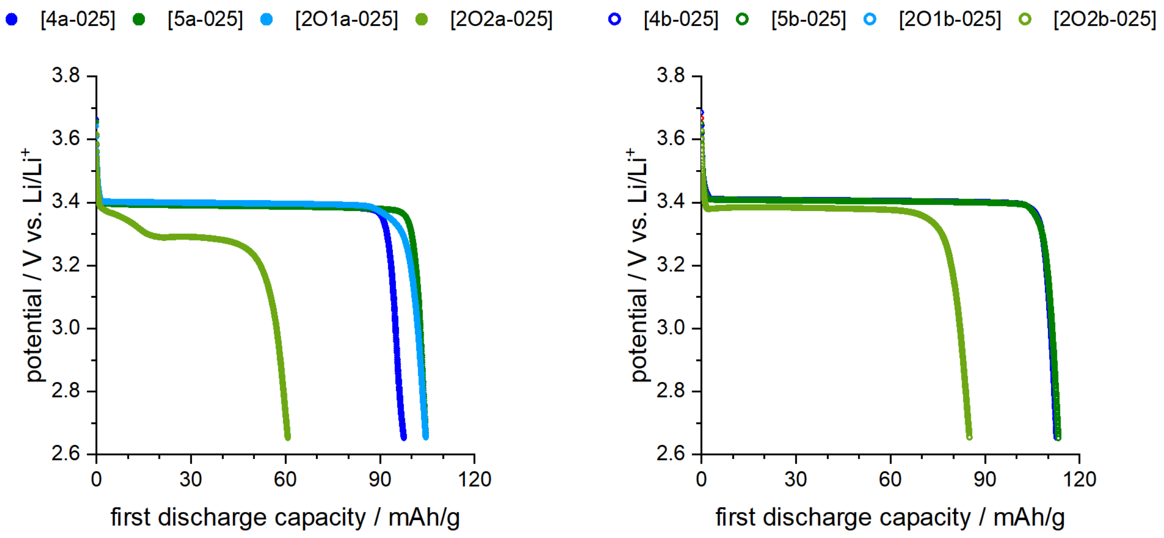

(a) (**b**)

Figure S29. Cycle test (half cell). In Fig. S29b, both mixtures ([4b-025] and [5b-025]) show almost the same behavior. The cell including mixture [201-025b] could not be discharged in a meaningful manner.

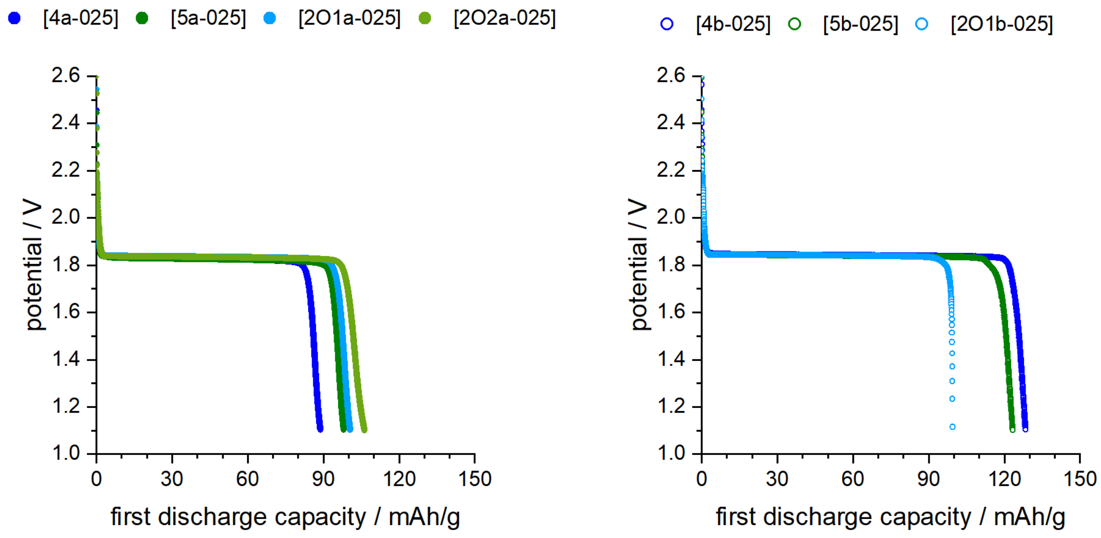

(a) (**b**)

Figure S30. Cycle test (full cell).

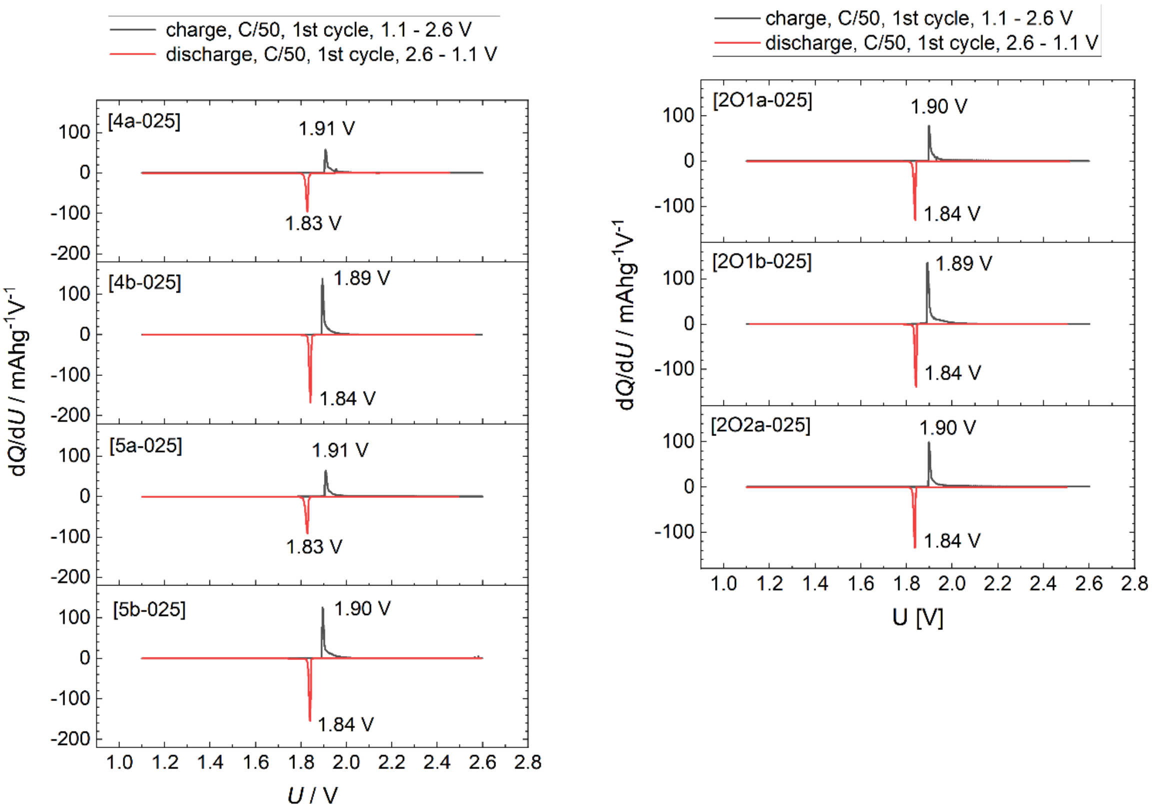

(a) (**b**)

Figure S31. dQ/dU during fist cycle for measuring the overpotential

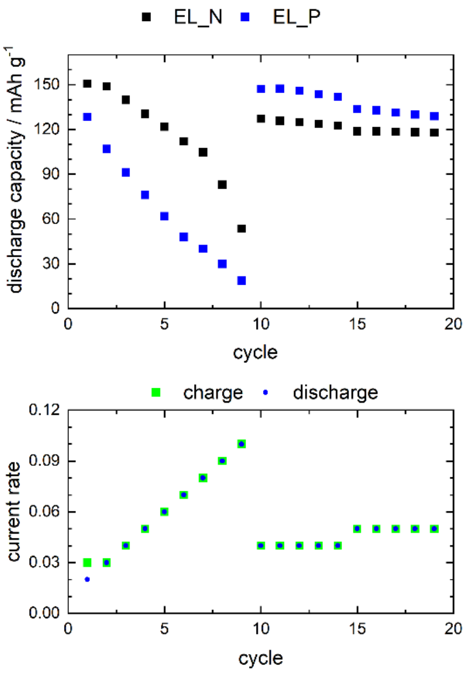

Figure S32. Comparison of ammonium and phosphonium based electrolytes: Shown are the cycle performance of two coin cells for each electrolyte mixture in half cell configuration against Li metal: EL_N: [N111(2O1)][TFSI] + saturated lithium difluoro(oxalate) borate (approx. 0.6 mol kg^{-1}); EL_P: [P111(2O1)][TFSI] + saturated lithium difluoro(oxalate) borate (approx. 0.5 mol kg^{-1}). Cycle 1-9 was done at T = 40 °C; cycle 10-19 was done at T = 60 °C.
